# Confusion2Vec: towards enriching vector space word representations with representational ambiguities

**DOI:** 10.7717/peerj-cs.195

**Published:** 2019-06-10

**Authors:** Prashanth Gurunath Shivakumar, Panayiotis Georgiou

**Affiliations:** Electrical and Computer Engineering, University of Southern California, Los Angeles, CA, USA

**Keywords:** Confusion2vec, Word2vec, Embeddings, Word representations, Confusion networks, ASR output representations, Lexical representational ambiguity

## Abstract

Word vector representations are a crucial part of natural language processing (NLP) and human computer interaction. In this paper, we propose a novel word vector representation, Confusion2Vec, motivated from the human speech production and perception that encodes representational ambiguity. Humans employ both acoustic similarity cues and contextual cues to decode information and we focus on a model that incorporates both sources of information. The representational ambiguity of acoustics, which manifests itself in word confusions, is often resolved by both humans and machines through contextual cues. A range of representational ambiguities can emerge in various domains further to acoustic perception, such as morphological transformations, word segmentation, paraphrasing for NLP tasks like machine translation, etc. In this work, we present a case study in application to automatic speech recognition (ASR) task, where the word representational ambiguities/confusions are related to acoustic similarity. We present several techniques to train an acoustic perceptual similarity representation ambiguity. We term this Confusion2Vec and learn on unsupervised-generated data from ASR confusion networks or lattice-like structures. Appropriate evaluations for the Confusion2Vec are formulated for gauging acoustic similarity in addition to semantic–syntactic and word similarity evaluations. The Confusion2Vec is able to model word confusions efficiently, without compromising on the semantic-syntactic word relations, thus effectively enriching the word vector space with extra task relevant ambiguity information. We provide an intuitive exploration of the two-dimensional Confusion2Vec space using principal component analysis of the embedding and relate to semantic relationships, syntactic relationships, and acoustic relationships. We show through this that the new space preserves the semantic/syntactic relationships while robustly encoding acoustic similarities. The potential of the new vector representation and its ability in the utilization of uncertainty information associated with the lattice is demonstrated through small examples relating to the task of ASR error correction.

## Introduction

Decoding human language is challenging for machines. It involves estimation of efficient, meaningful representation of words. Machines represent the words in the form of real vectors and the language as a vector space. Vector space representations of language have applications spanning natural language processing (NLP) and human computer interaction fields. More specifically, word embeddings can act as features for machine translation, automatic speech recognition (ASR), document topic classification, information retrieval, sentiment classification, emotion recognition, behavior recognition, question answering, etc.

Early work employed words as the fundamental unit of feature representation. This could be thought of as each word representing an orthogonal vector in a *n*-dimensional vector space of language with *n*-words (often referred to as one-hot representation). Such a representation, due to the inherent orthogonality, lacks crucial information regarding inter–word relationships such as similarity. Several techniques found using co-occurrence information of words are a better feature representation (Ex: *n*-gram language modeling).

Subsequent studies introduced few matrix factorization based techniques to estimate a more efficient, reduced dimensional vector space based on word co-occurrence information. Latent semantic analysis (LSA) assumes an underlying vector space spanned by orthogonal set of latent variables closely associated with the semantics/meanings of the particular language. The dimension of this vector space is much smaller than the one-hot representation (Deerwester et al., 1990). LSA was proposed initially for information retrieval and indexing, but soon gained popularity for other NLP tasks. Hofmann (1999) proposed probabilistic LSA replacing the co-occurrence information by a statistical class based model leading to better vector space representations.

Another popular matrix factorization method, the latent dirichlet allocation assumes a generative statistical model where the documents are characterized as a mixture of latent variables representing topics which are described by word distributions (Blei, Ng & Jordan, 2003).

Recently, neural networks have gained popularity. They often outperform the *n*-gram models (Bengio et al., 2003; Mikolov et al., 2010) and enable estimation of more complex models incorporating much larger data than before. Various neural network based vector space estimation of words were proposed. Bengio et al. (2003) proposed feed-forward neural network based language models which jointly learned the distributed word representation along with the probability distribution associated with the representation. Estimating a reduced dimension continuous word representation allows for efficient probability modeling, thereby resulting in much lower perplexity compared to an *n*-gram model. Recurrent neural network based language models, with inherent memory, allowed for the exploitation of much longer context, providing further improvements compared to feed forward neural networks (Mikolov et al., 2010).

Mikolov et al. (2013a) proposes a new technique of estimating vector representation (popularly termed word2vec) which showed promising results in preserving the semantic and syntactic relationships between words. Two novel architectures based on simple log-linear modeling (i) continuous skip-gram and (ii) continuous bag-of-words are introduced. Both the models are trained to model local context of word occurrences. The continuous skip-gram model predicts surrounding words given the current word. Whereas, the continuous bag-of-words model predicts the current word given its context. The task evaluation is based on answering various analogy questions testing semantic and syntactic word relationships. Several training optimizations and tips were proposed to further improve the estimation of the vector space by Mikolov et al. (2013c) and Mnih & Kavukcuoglu (2013). Such efficient representation of words directly influences the performance of NLP tasks like sentiment classification (Kim, 2014), part-of-speech tagging (Ling et al., 2015), text classification (Lilleberg, Zhu & Zhang, 2015; Joulin et al., 2016), document categorization (Xing et al., 2014), and many more.

Subsequent research efforts on extending word2vec involve expanding the word representation to phrases (Mikolov et al., 2013c), sentences and documents (Le & Mikolov, 2014). Similarly, training for contexts derived from the syntactic dependencies of a word is shown to produce useful representations (Levy & Goldberg, 2014). Using morphemes for word representations can enrich the vector space and provide gains especially for unknown, rarely occurring, complex words, and morphologically rich languages (Luong, Socher & Manning, 2013; Botha & Blunsom, 2014; Qiu et al., 2014; Cotterell & Schütze, 2015; Soricut & Och, 2015). Likewise, incorporating sub-word representations of words for the estimation of vector space is beneficial (Bojanowski et al., 2017). Similar studies using characters of words have also been tried (Chen et al., 2015). Yin & Schütze (2016) explored ensemble techniques for exploiting complementary information over multiple word vector spaces. Studies by Mikolov, Le & Sutskever (2013b) and Faruqui & Dyer (2014) demonstrate that vector space representations are extremely useful in extending the model from one language to another (or multi-lingual extensions) since the semantic relations between words are invariant across languages.

Some have tried to combine the advantages from both matrix factorization based techniques and local-context word2vec models. Pennington, Socher & Manning (2014) proposes global log-bilinear model for modeling global statistical information as in the case of global matrix factorization techniques along with the local context information as in the case of word2vec.

**Figure 1:**
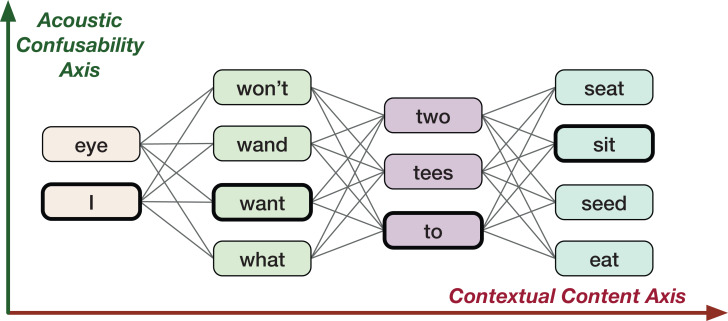
An example confusion network for ground-truth utterance “I want to sit.”

The goal of this study is to come up with a new vector space representation for words which incorporates the uncertainty information in the form of word confusions present in lattice like structures (e.g., confusion networks). Here, the word confusions refers to any word level ambiguities resultant of perception confusability or any algorithms such as machine translation, ASR etc. For example, acoustically confusable words in ASR lattices: “two” and “to” (see Fig. 1). A word lattice is a compact representation (directed acyclic weighted graphs) of different word sequences that are likely possible. A confusion network is a special type of lattice, where each word sequence is made to pass through each node of the graph. The lattices and confusion networks embed word confusion information. The study takes motivation from human perception, that is, the ability of humans to decode information based on two fairly independent information streams (see section “Human Speech Production, Perception and Hearing” for examples): (i) linguistic context (modeled by word2vec like word vector representations), and (ii) acoustic confusability (relating to phonology).

The present word vector representations like word2vec only incorporate the contextual confusability during modeling. However, in order to handle confusability and to decode human language/speech successfully, there is a need to model both the dimensions. Although primarily, the motivation is derived from human speech and perception, the confusions are not constrained to acoustics and can be extended to any confusions parallel to the linguistic contexts, for example, confusions present in lattices. Most of the machine learning algorithms output predictions as a probability measure. This uncertainty information stream can be expressed in the form of a lattice or a confusion network temporally, and is often found to contain useful information for subsequent processing and analysis. The scope of this work is to introduce a complementary (ideally orthogonal) subspace in addition to the underlying word vector space representation captured by word2vec. This new subspace captures the word confusions orthogonal to the syntactic and semantics of the language. We propose Confusion2Vec vector space operating on lattice like structures, specifically word confusion networks. We introduce several training configurations and evaluate their effectiveness. We also formulate appropriate evaluation criterion to assess the performance of each orthogonal subspaces, first independently and then jointly. Analysis of the proposed word vector space representation is carried out.

The rest of the paper is organized as follows. Motivation for Confusion2vec, that is, the need to model word-confusions for word embeddings, is provided through means of human speech and perception, machine learning, and through potential applications in the section “Motivation”. A particular case study is chosen and the problem is formulated in the section “Case Study: Application to Automatic Speech Recognition”. In the section “Proposed Models”, different training configurations for efficient estimation of word embeddings are proposed. Additional tuning schemes for the proposed Confusion2vec models are presented in the section “Training Schemes”. Evaluation criterion formulation and evaluation database creation is presented in the section “Evaluation Methods”. Experimental setup and baseline system is described in the section “Data and Experimental Setup”. Results are tabulated and discussed in the section “Results”. Word vector space analysis is performed and findings are presented in the section “Vector Space Analysis”. Section “Discussion” discusses with the help of few toy examples, the benefits of the Confusion2vec embeddings for the task of ASR error correction. Section “Conclusion” draws the conclusion of the study and finally the future research directions are discussed in the section “Future Work”.

## Motivation

One efficient way to represent words as vectors is to represent them in a space that preserves the semantic and syntactic relations between the words in the language. Word2vec describes a technique to achieve such a representation by trying to predict the current word from its local context (or vice-versa) over a large text corpora. The estimated word vectors are shown to encode efficient syntactic-semantic language information. In this work, we propose a new vector space for word representation which incorporates various forms of word confusion information in addition to the semantic and syntactic information. The new vector space is inspired and motivated from the following factors from human speech production and perception and machine learning.

### Human speech production, perception, and hearing

In our everyday interactions, confusability can often result in the need for context to decode the underlying words.

“Please_____ a seat.”      (Example 1)

In Example 1, the missing word could be guessed from its context and narrowed down to either “have” or “take.” This context information is modeled through language models. More complex models such as word2vec also use the contextual information to model word vector representations.

On the other hand, confusability can also originate from other sources such as acoustic representations.

“I want to seat”        (Example 2)

In Example 2, the underlined word is mispronounced/misheard, and grammatically incorrect. In this case, considering the context there exists a lot of possible correct substitutions for the word “seat” and hence the context is less useful. The acoustic construct of the word “seat” can present additional information in terms of acoustic alternatives/similarity, such as “sit” and “seed.”

“I want to s—”        (Example 3)

Similarly in Example 3, the underlined word is incomplete. The acoustic confusability information can be useful in the above case of broken words. Thus, since the confusability is acoustic, purely lexical vector representations like word2vec fail to encode or capture it. In this work, we propose to additionally encode the word (acoustic) confusability information to learn a better word embedding. Although the motivation is specific to acoustics in this case, it could be extended to other inherent sources of word-confusions spanning various machine learning applications.

### Machine learning algorithms

Most of the machine learning algorithms output hypothesis as a probability measure. Such a hypothesis could be represented in the form of a lattice, confusion network or *n*-best lists. It is often useful to consider the uncertainty associated with the hypothesis for subsequent processing and analysis (see section “Potential Applications”). The uncertainty information is often, orthogonal to the contextual dimension and is specific to the task attempted by the machine learning algorithms.

Along this direction, recently, there have been several efforts concentrated on introducing lattice information into the neural network architecture. Initially, Tree-LSTM was proposed enabling tree-structured network topologies to be inputted to the RNNs (Tai, Socher & Manning, 2015), which could be adapted and applied to lattices (Sperber et al., 2017). LatticeRNN was proposed for processing word level lattices for ASR (Ladhak et al., 2016). Lattice based gated recurrent units (Su et al., 2017) and lattice-to-sequence models (Tan et al., 2018) were proposed for reading word lattice as input, specifically a lattice with tokenization alternatives for machine translation models. LatticeLSTM was adopted for lattice-to-sequence model incorporating lattice scores for the task of speech translation by Sperber et al. (2017). Buckman & Neubig (2018) proposed Neural lattice language models which enables to incorporate many possible meanings for words and phrases (paraphrase alternatives).

Thus, a vector space representation capable of embedding relevant uncertainty information in the form of word confusions present in lattice-like structures or confusion networks along with the semantic and syntactic can be potentially superior to word2vec space.

## Case Study: Application to Automatic Speech Recognition

In this work, we consider the ASR task as a case study to demonstrate the effectiveness of the proposed Confusion2vec model in modeling acoustic word-confusability. However, the technique can be adopted for a lattice or confusion network output from potentially any algorithm to capture various patterns as discussed in the section “Potential Applications,” in which case the confusion-subspace (vertical ambiguity in Fig. 1), is no longer constrained to acoustic word-confusions.

An ASR lattice contains multiple paths over acoustically similar words. A lattice could be transformed and represented as a linear graph forcing every path to pass through all the nodes (Xue & Zhao, 2005; Mangu, Brill & Stolcke, 2000). Such a linear graph is referred to as a confusion network. Figure 1 shows a sample confusion network output by ASR for the ground truth “I want to sit.” The confusion network could be viewed along two fundamental dimensions of information (see Fig. 1): (i) Contextual axis—sequential structure of a sentence, (ii) Acoustic axis—similarly sounding word alternatives. Traditional word vector representations such as word2vec only model the contextual information (the horizontal (red) direction in Fig. 1). The word confusions, for example, the acoustic contextualization as in Fig. 1 (the vertical (green) direction in Fig. 1) is not encoded. We propose to additionally capture the co-occurrence information along the acoustic axis orthogonal to the word2vec. This is the main focus of our work, that is, to jointly learn the vertical, word-confusion context and the horizontal, semantic and syntactic context. In other words, we hypothesize to derive relationships between the semantics and syntaxes of language and the word-confusions (acoustic-confusion).

### Related work

Bengio & Heigold (2014) trained a continuous word embedding of acoustically alike words (using *n*-gram feature representation of words) to replace the state space models (Hidden Markov Models, HMMs), decision trees, and lexicons of an ASR. Through the use of such an embedding and lattice re-scoring technique demonstrated improvements in word error rates of ASR. The embeddings are also shown to be useful in application to the task of ASR error detection by Ghannay et al. (2016). A few evaluation strategies are also devised to evaluate phonetic and orthographic similarity of words. Additionally, there have been studies concentrating on estimating word embeddings from acoustics (Kamper, Wang & Livescu, 2016; Chung et al., 2016; Levin et al., 2013; He, Wang & Livescu, 2016) with evaluations based on acoustic similarity measures. Parallely, word2vec like word embeddings have been used successfully to improve ASR Error detection performance (Ghannay, Estève & Camelin, 2015a; Ghannay et al., 2015b). We believe the proposed exploitation of both information sources, that is, acoustic relations and linguistic relations (semantics and syntaxes) will be beneficial in ASR and error detection, correction tasks. The proposed confusion2vec operates on the lattice output of the ASR in contrast to the work on acoustic word embeddings (Kamper, Wang & Livescu, 2016; Chung et al., 2016; Levin et al., 2013; He, Wang & Livescu, 2016) which is directly trained on audio. The proposed Confusion2vec differs from works by Bengio & Heigold (2014) and Ghannay et al. (2016), which also utilize audio data with the hypothesis that the layer right below softmax layer of a deep end-to-end ASR contains acoustic similarity information of words. Confusion2vec can also be potentially trained without an ASR, on artificially generated data, emulating an ASR (Tan et al., 2010; Sagae et al., 2012; Celebi et al., 2012; Kurata, Itoh & Nishimura, 2011; Dikici, Celebi & Saraçlar, 2012; Xu, Roark & Khudanpur, 2012). Thus, Confusion2vec can potentially be trained in a completely unsupervised manner and with appropriate model parameterization incorporate various degrees of acoustic confusability, for example, stemming from noise or speaker conditions.

Further, in contrast to the prior works on lattice encoding RNNs (Tai, Socher & Manning, 2015; Sperber et al., 2017; Ladhak et al., 2016; Su et al., 2017; Tan et al., 2018; Buckman & Neubig, 2018), which concentrate on incorporating the uncertainty information embedded in the word lattices by modifying the input architecture for recurrent neural network, we propose to introduce the ambiguity information from the lattices to the word embedding explicitly. We expect similar advantages as with lattice encoding RNNs in using the pre-trained confusion2vec embedding toward various tasks like ASR, Machine translation etc. Moreover, our architecture doesn’t require memory which has significant advantages in terms of training complexity. We propose to train the embedding in a similar way to word2vec models (Mikolov et al., 2013a). All the well studied previous efforts toward optimization of training such models (Mikolov et al., 2013c; Mnih & Kavukcuoglu, 2013), should apply to our proposed model.

## Proposed Models

In this section, we propose four training schemes for Confusion2Vec. The training schemes are based on the word2vec model. Word2vec work (Mikolov et al., 2013a) proposed log-linear models, that is, a neural network consisting of a single linear layer (projection matrix) without non-linearity. These models have significant advantages in training complexity. Mikolov et al. (2013a) found the skip-gram model to be superior to the bag-of-word model in a semantic-syntactic analogy task. Hence, we only employ the skip-gram configuration in this work. Appropriately, the skip-gram word2vec model is also adopted as the baseline for this work. The choice of the skip-gram modeling in this work is mainly based on its popularity, ease of implementation, low complexity, and being a well-proven technique in the community. However, we strongly believe the proposed concept (introducing word ambiguity information) is independent of the modeling technique itself and should translate to relatively newer techniques like GloVe Pennington, Socher & Manning (2014) and fastText Bojanowski et al. (2017).

### Top-confusion training—C2V-1

**Figure 2:**
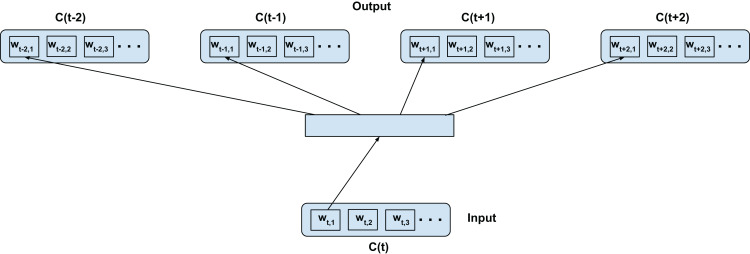
Top-confusion2vec training scheme for confusion networks. *C*(*t*) is a unit word confusion in the confusion network at a time-stamp *t*, that is, *C*(*t*) represents a set of arcs between two adjacent nodes of a confusion network, representing a set of confusable words. *w_t,i_* is the *i*th most probable word in the confusion *C*(*t*). Word confusions are sorted in decreasing order of their posterior probability: *P*(*w*_*t*,1_) > *P*(*w*_*t*,2_) > *P*(*w*_*t*,3_)….

We adapt the word2vec contextual modeling to operate on the confusion network (in our case confusion network of an ASR). Figure 2 shows the training configuration of the skip-gram word2vec model on the confusion network. The top-confusion model considers the context of only the top hypothesis of the confusion network (single path) for training. For clarity we call this the C2V-1 model since it’s using only the 1 top hypothesis. The words *w_t_*_−2,1_, *w_t_*_−1,1_, *w_t_*_+1,1_, and *w_t_*_+2,1_ (i.e., the most probable words in the confusions *C*(*t*−2), *C*(*t*−1), *C*(*t*+1), and *C*(*t*+2), respectively) are predicted from *w_t_*_,1_ (i.e., the most probable word in *C*(*t*)) for a skip-window of 2 as depicted in Fig. 2. The top hypothesis typically consists of noisy transformations of the reference ground-truth (Note: the confusion network will inherently introduce additional paths to the lattice). In the case of a confusion network of an ASR, the noisy transformations correspond to acoustic word confusions. Thus, the top-confusion model implicitly captures word confusions (co-occurring within the context of the skip-window).

### Intra-confusion training—C2V-a

**Figure 3:**
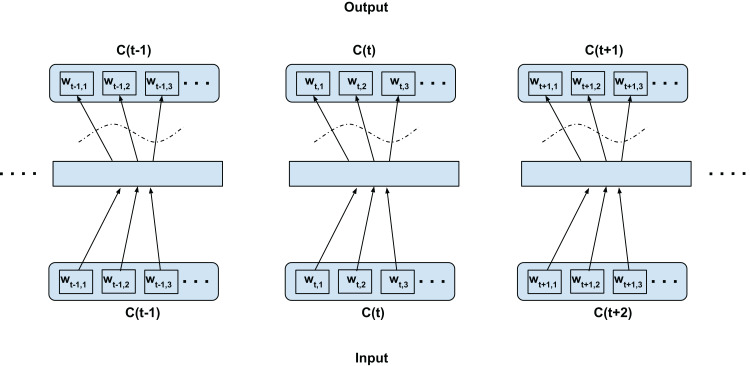
Proposed intra-confusion training scheme for confusion networks. *C*(*t*) is a unit word confusion in the confusion network at a time-stamp *t*, that is, *C*(*t*) represents a set of arcs between two adjacent nodes of a confusion network, representing a set of confusable words. *w_t,i_* is the *i*th most probable word in the confusion *C*(*t*). Word confusions are sorted in decreasing order of their posterior probability: *P*(*w*_*t*,1_) > *P*(*w*_*t*,2_) > *P*(*w*_*t*,3_)…. The dotted curved lines denote that the self-mapping is disallowed.

Next, we explore the direct adaptation of the skip-gram modeling but on the confusion dimension (i.e., considering word confusions as contexts) rather than the traditional sequential context. Figure 3 shows the training configuration over a confusion network. In short, every word is linked with every other alternate word in the confusion dimension (i.e., between set of confusable words) through the desired network (as opposed to the temporal context dimension in the word2vec training). For clarity, since this is only using acoustically alternate words, we call this the C2V-acoustics or C2V-a model for short. Note, we disallow any word being predicted from itself (this constrain is indicated with curved dotted lines in the figure). As depicted in the Fig. 3, the word *w_t,i_* (confusion context) is predicted from *w_t,j_* (current word), where *i* = 1,2,3…length(*C*(*t*)) and *j* ≠ *i*, for each *j* = 1,2,3…length(*C*(*t*)) for confusion *C*(*t*) ∀*t*. We expect such a model to capture inherent relations over the different word confusions. In the context of an ASR lattice, we expect it to capture intrinsic relations between similarly sounding words (acoustically similar). However, the model would fail to capture any semantic and syntactic relations associated with the language. The embedding obtained from this configuration can be fused (concatenated) with the traditional skip-gram word2vec embedding to form a new subspace representing both the independently trained subspaces. The number of training samples generated with this configuration is:

(1) $$\# {\rm{Samples}} = \sum\limits_{i = 1}^n {{D_i}} \times ({D_i} - 1)$$

where *n* is the number of time steps, *D_i_* is the number of confusions at the *i*th time step.

### Inter-confusion training—C2V-c

**Figure 4:**
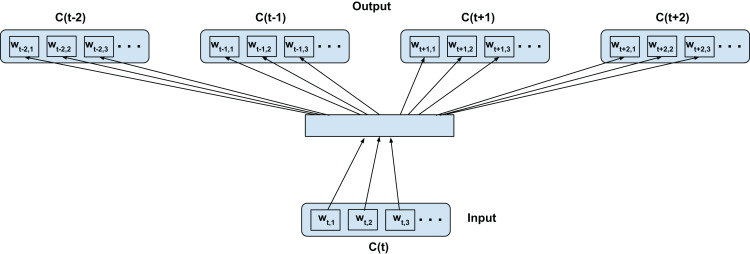
Proposed inter-confusion training scheme for confusion networks. *C*(*t*) is a unit word confusion in the confusion network at a time-stamp *t*, that is, *C*(*t*) represents a set of arcs between two adjacent nodes of a confusion network, representing a set of confusable words. *w_t,i_* is the *i*th most probable word in the confusion *C*(*t*). Word confusions are sorted in decreasing order of their posterior probability: *P*(*w*_*t*,1_) > *P*(*w*_*t*,2_) > *P*(*w*_*t*,3_)….

In this configuration, we propose to model both the linguistic contexts and the word confusion contexts simultaneously. Figure 4 illustrates the training configuration. Each word in the current confusion is predicted from each word from the succeeding and preceding confusions over a predefined local context. To elaborate, the words *w_t_*_−*t*′,*i*_ (context) are predicted from *w_t,j_* (current word) for *i* = 1,2,3…length(*C*(*t*−*t*′)), *j* = 1,2,3…length(*C*(*t*)), *t*′ ∈ 1,2,−1,−2 for skip-window of 2 for current confusion *C*(*t*)∀*t* as per Fig. 4. Since we assume the acoustic similarities for a word to be co-occurring, we expect to jointly model the co-occurrence of both the context and confusions. For clarity, since even the acoustic similarities are learned through context and not direct acoustic mapping, as in the Intra-confusion case, we call the inter-confusion training C2V-context or C2V-c for short.

This also has the additional benefit of generating more training samples than the intra-confusion training. The number of training samples generated is given by:

(2) $$\# {\rm{Samples}} = \sum\limits_{i = 1}^n \sum\limits_{{\scriptstyle j = i - {S_w} \atop \scriptstyle j \ne i} }^{i + {S_w}} {D_i} \times {D_j}$$

where *n* is the total number of time steps, *D_i_* is the number of word confusions at the *i*^th^ time step, *S_w_* is the skip-window size (i.e., sample *S_w_* words from history and *S_w_* words from the future context of current word). Inter-confusion training can be viewed as an extension of top-confusion training where the skip-gram modeling is applied to all possible paths through the confusion network.

### Hybrid intra-inter confusion training—C2V-*

**Figure 5:**
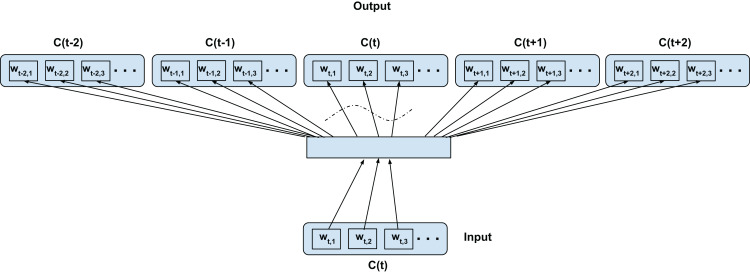
Proposed hybrid-confusion training scheme for confusion networks. *C*(*t*) is a unit word confusion in the confusion network at a time-stamp *t*, that is, *C*(*t*) represents a set of arcs between two adjacent nodes of a confusion network, representing a set of confusable words. *w_t,i_* is the *i*th most probable word in the confusion *C*(*t*). Word confusions are sorted in decreasing order of their posterior probability: *P*(*w*_*t*,1_) > *P*(*w*_*t*,2_) > *P*(*w*_*t*,3_)…. The dotted curved lines denote that the self-mapping is disallowed.

Finally, we merge both the intra-confusion and inter-confusion training. For clarity we call this model the C2V-* since it combines all the previous cases. This can be seen as a super-set of top-confusion, inter-confusion and intra-confusion training configurations. Figure 5 illustrates the training configuration. The words *w_t_*_−*t*′,*i*_ (context) are predicted from *w_t,j_* (current word) for *i* = 1,2,3…length(*C*(*t*−*t*′)), *j* = 1,2,3…length(*C*(*t*)), *t*′ ∈ 1,2,0,−1,−2 such that if *t*′ = 0 then *i* ≠ *j*; for skip-window of 2 for current confusion *C*(*t*)∀*t* as depicted in Fig. 5. We simply add the combination of training samples from the above two proposed techniques (i.e., the number of samples is the sum of Eqs. (1) and (2)).

## Training Schemes

### Model initialization/pre-training

**Figure 6:**
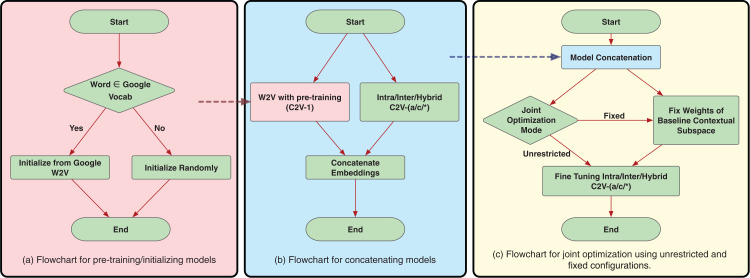
Flowcharts for proposed training schemes.

Very often, it has been found that better model initializations lead to better model convergence (Erhan et al., 2010). This is more significant in the case of under-represented words. Moreover, for training the word confusion mappings, it would benefit to build upon the contextual word embeddings, since our final goal is in conjunction with both contextual and confusion information. Hence, we experiment initializing all our models with the original Google’s word2vec model (https://code.google.com/archive/p/word2vec/) trained on Google News dataset with 100 billion words as described by Mikolov et al. (2013c). Pre-training rules are explained in the flowchart in Fig. 6A. For the words present in the Google’s word2vec vocabulary, we directly initialize the embeddings with word2vec. The embeddings for rest of the words are randomly initialized following uniform distribution.

### Model concatenation

The hypothesis with model concatenation is that the two subspaces, one representing the contextual subspace (word2vec), and the other capturing the confusion subspace can be both trained independently and concatenated to give a new vector space which manifests both the information and hence a potentially useful vector word representation. Flowchart for model concatenation is shown in Fig. 6B. The model concatenation can be mathematically represented as:

(3) $${\rm{NE}}{{\rm{W}}_{n \times {e_1} + {e_2}}} = \left[ {W2{V_{n \times {e_1}}}\quad C2{V_{n \times {e_2}}}} \right]$$

where NEW is the new concatenated vector space of dimensions *n* × *e*_1_ + *e*_2_, and *n* is the vocabulary size, *e*_1_ and *e*_2_ are the embedding sizes of *W*2*V* and *C*2*V* subspaces, respectively.

### Joint optimization

Further to the model concatenation scheme, one could fine-tune the new vector space representation to better optimize to the task criterion (fine-tuning involves re-training end-to-end with a relatively lower learning rate than usual). This could be viewed as a case of relaxing the strict independence between two subspaces as in the case of model concatenation. The fine-tuning itself could be either of the aforementioned proposed techniques. We specifically try two configurations of joint optimization.

#### i. Fixed contextual subspace

In this configuration, we fix the contextual (word2vec) subspace and fine-tune only the confusion subspace. Since the word2vec already provides robust contextual representation, any fine-tuning on contextual space could possibly lead to sub-optimal state. Keeping the word2vec subspace fixed also allows the model to concentrate more specifically toward the confusion since the fixed subspace compensates for all the contextual mappings during training. This allows us to constrain the updatable parameters during joint optimization. It also allows for the possibility to directly use available word2vec models without modifications. The flowchart for the fixed contextual subspace joint optimization is displayed in Fig. 6C.

#### ii. Unrestricted

In this configuration, we optimize both the subspaces, that is, the contextual (word2vec) and the confusion subspaces. The hypothesis is the fine-tuning allows the two subspaces to interact to achieve the best possible representation. The flowchart for the unrestricted joint optimization is displayed in Fig. 6C.

## Evaluation Methods

Prior literature suggests, there are two prominent ways for evaluating the vector space representation of words. One is based on semantic and syntactic analogy task as introduced by Mikolov et al. (2013a). The other common approach has been to assess the word similarities by computing the rank-correlation (Spearman’s correlation) on human annotated word similarity databases (Schnabel et al., 2015) like WordSim-353 (Finkelstein et al., 2001). Although the two evaluations can judge the vector representations of words efficiently for semantics and syntax of a language, we need to device an evaluation criteria for the word confusions, specifically for our case scenario—the acoustic confusions of words. For this, we formulate evaluations for acoustic confusions parallel to the analogy task and the word similarity task.

### Analogy tasks

#### Semantic and syntactic analogy task

Mikolov et al. (2013a) introduced an analogy task for evaluating the vector space representation of words. The task was based on the intuition that the words, say “king” is similar to “man” in the same sense as the “queen” is to “woman” and thus relies on answering questions relating to such analogies by performing algebraic operations on word representations. For example, the analogy is correct if the vector(“woman”) is most similar to vector(“king”) − vector(“man”) + vector(“queen”). The analogy question test set is designed to test both syntactic and semantic word relationships. The test set contains five types of semantic questions (8,869 questions) and nine types of syntactic questions (10,675 questions). Finally, the efficiency of the vector representation is measured using the accuracy achieved on the analogy test set. We employ this for testing the semantic and syntactic (contextual axis as in terms of Fig. 1) relationship inherent in the vector space.

#### Acoustic analogy task

The primary purpose of the acoustic analogy task is to independently gauge the acoustic similarity information captured by the embedding model irrespective of the inherent semantic and syntactic linguistic information. Adopting a similar idea and extending the same for evaluation of word confusions, we formulate the acoustic confusion analogy task (vertical context test as in terms of Fig. 1) as follows. For similar sounding word pairs, “see” & “sea” and “red” & “read,” the word vector “see” is similar to “sea” in the same sense as the word “red” is to “read.” We set up an acoustic analogy question set on acoustically similar sounding words, more specifically homophones. Table 1 lists a few examples from our data set. A detailed description of the creation of dataset is presented in the section “Creation of evaluation datasets.”

**Table 1:** Few examples from acoustic analogy task test-set.

Word pair 1		Word pair 2	
I’d	Eyed	Phi	Fie
Seedar	Cedar	Rued	Rude
Air	Aire	Spade	Spayed
Scent	Cent	Vile	Vial
Cirrus	Cirrous	Sold	Soled
Curser	Cursor	Pendant	Pendent
Sensor	Censor	Straight	Strait

#### Semantic and syntactic–acoustic analogy task

Further, rather than evaluating the semantic-syntactic tasks and the acoustic analogy tasks independently, we could test for both together. Intuitively, the word vectors in each of the two subspaces should interact together. We would expect for an analogy, “see”-“saw”:“take”-“took,” the word “see” has a homophone alternative in “sea,” thus there is a possibility of the word “see” being confused with “sea” in the new vector space. Thus an algebraic operation such as vector(“see”) − vector(“saw”) + vector(“take”) should be similar to vector(“took”) as before. Moreover, the vector(“sea”) − vector(“saw”) + vector(“take”) should also be similar to vector(“took”). This is because we expect the vector(“sea”) to be similar to vector(“see”) under the acoustic subspace. We also take into account the more challenging possibility of more than one homophone word substitution. For example, vector(“see”) − vector(“saw”) + vector(“allow”) is similar to vector(“allowed”), vector(“aloud”), and vector(“sea”) − vector(“saw”) + vector(“allow”). The hypothesis is that to come up with such a representation the system should jointly model both the language semantic-syntactic relations and the acoustic word similarity relations between words. The task is designed to test semantic–acoustic relations and the syntactic–acoustic relationships. In other words, in terms of Fig. 1, the task evaluates both the horizontal and vertical context together. A few examples of this task is listed in Table 2. In the section “Creation of evaluation datasets” details the creation of the database.

**Table 2:** Few examples from Semantic&Syntactic—acoustic analogy task test set.

Type of relationship	Word pair 1		Word pair 2	
Currency	India	Rupee	Korea	One (Won)
	Canada	Dollar	Denmark	Krona (Krone)
	Japan	Yen	Sweden	Krone (Krona)
Family	Buoy (Boy)	Girl	Brother	Sister
	Boy	Girl	King	Quean (Queen)
	Boy	Girl	Sun (Son)	Daughter
Adjective-to-adverb	Calm	Calmly	Sloe (Slow)	Slowly
Opposite	Aware	Unaware	Possible	Impassible (Impossible)
Comparative	Bad	Worse	High	Hire (Higher)
Superlative	Bad	Worst	Grate (Great)	Greatest
Present participle	Dance	Dancing	Rite (Write)	Writing
Past tense	Dancing	Danced	Flying	Flu (Flew)
Plural	Banana	Bananas	Burred (Bird)	Birds
Plural verbs	Decrease	Decreases	Fined (Find)	Finds
Multiple homophone substitutions	Wright (Write)	Writes	Sea (See)	Sees
	Rowed (Road)	Roads	I (Eye)	Ayes (Eyes)
	Si (See)	Seize (Sees)	Right (Write)	Writes

**Note:**

The words in the parenthesis are the original ones as in the analogy test set (Mikolov et al., 2013a) which have been replaced by their homophone alternatives.

### Similarity ratings

#### Word similarity ratings

Along with the analogy task the word similarity task (Finkelstein et al., 2001) has been popular to evaluate the quality of word vector representations in the NLP community (Pennington, Socher & Manning, 2014; Luong, Socher & Manning, 2013; Huang et al., 2012; Schnabel et al., 2015). In this work, we employ the WordSim-353 dataset (Finkelstein et al., 2001) for the word similarity task. The dataset has a set of 353 word pairs with a diverse range of human annotated scores relating to the similarity/dissimilarity of the two words. The rank-order correlation (Spearman correlation) between the human annotated scores and the cosine similarity of word vectors is computed. Higher correlation corresponds to better preservation of word similarity order represented by the word vectors, and hence better quality of the embedding vector space.

#### Acoustic similarity ratings

Employing a similar analogous idea to word similarity ratings and extending it to reflect the quality of word confusions, we formulate an acoustic word similarity task. The attempt is to have word pairs scored similar to as in WordSim-353 database, but with the scores reflecting the acoustic similarity. Table 3 lists a few randomly picked examples from our dataset. The dataset generation is described in the section “Creation of evaluation datasets”.

**Table 3:** Examples of acoustic similarity ratings.

Word1	Word2	Acoustic rating	WordSim353
I	Eye	1.0	–
Adolescence	Adolescents	0.9	–
Allusion	Illusion	0.83	–
Sewer	Sower	0.66	–
Fighting	Defeating	0.57	7.41
Day	Dawn	0.33	7.53
Weather	Forecast	0.0	8.34

**Notes:**

Acoustic rating: 1.0 = Identically sounding, 0.0 = Highly acoustically dissimilar.

WordSim353: 10.0 = High word similarity, 0.0 = Low word similarity.

Word pairs not present in WordSim353 is denoted by “–”.

## Data and Experimental Setup

### Database

We employ Fisher English Training Part 1, Speech (LDC2004S13) and Fisher English Training Part 2, Speech (LDC2005S13) corpora (Cieri, Miller & Walker, 2004) for training the ASR. The corpora consists of approximately 1,915 h of telephone conversational speech data sampled at 8 kHz. A total of 11,972 speakers were involved in the recordings. The speech corpora is split into three speaker disjoint subsets for training, development and testing for ASR modeling purposes. A subset of the speech data containing approximately 1,905 h were segmented into 1,871,731 utterances to train the ASR. Both the development set and the test set consists of 5,000 utterances worth 5 h of speech data each. The transcripts contain approximately 20.8 million word tokens with 42,150 unique entries.

### Experimental setup

#### Automatic speech recognition

KALDI toolkit is employed for training the ASR (Povey et al., 2011). A hybrid DNN-HMM based acoustic model is trained on high resolution (40 dimensional) mel frequency cepstral coefficients along with *i*-vector features to provide speaker and channel information for robust modeling. The Carnegie Mellon University (CMU) pronunciation dictionary (Weide, 1998) is pruned to corpora’s vocabulary and is used as a lexicon for the ASR. A trigram language model is trained on the transcripts of the training subset data. The ASR system achieves a word error rates of 16.57% on the development and 18.12% on the test datasets. The decoded lattice is used to generate confusion network based on minimum Bayes risk criterion (Xu et al., 2011). The ASR output transcriptions resulted in a vocabulary size of 41,274 unique word tokens.

#### Confusion2Vec

For training the Confusion2Vec, the training subset of the Fisher corpora is used. The total number of tokens resulting from the multiple paths over the confusion network is approximately 69.5 million words, that is, an average of 3.34 alternative word confusions present for each word in the confusion network. A minimum frequency threshold of 5 is set to prune the rarely occurring tokens from the vocabulary, which resulted in the reduction of the vocabulary size from 41,274 to 32,848. Further, we also subsample the word tokens as suggested by Mikolov et al. (2013c) which was shown to be helpful. Both the frequency thresholding and the downsampling resulted in a reduction of word tokens from 69.5 million words to approximately 33.9 million words. The Confusion2Vec and Word2Vec are trained using the Tensorflow toolkit (Abadi et al., 2016). Negative Sampling objective is used for training as suggested for better efficiency (Mikolov et al., 2013c). For the skip-gram training, the batch-size of 256 was chosen and 64 negative samples were used for computing the negative sampling loss. The skip-window was set to 4 and was trained for a total of 15 epochs. The parameters were chosen to provide optimal performance with traditional word2vec embeddings, evaluating for word analogy task, for the size of our database. During fine-tuning, the model was trained with a reduced learning rate and with other parameters unchanged. All the above parameters were fixed for consistent and fair comparison.

### Creation of evaluation datasets

#### Acoustic analogy task

We collected a list of homophones in English (http://homophonelist.com/homophones-list/ Accessed: 2018-04-30), and created all possible combinations of pairs of acoustic confusion analogies. For homophones with more than two words, we list all possible confusion pairs. Few examples from the dataset are listed in Table 1. We emphasize that the consideration of only homophones in the creation of the dataset is a strict and a difficult task to solve, since the ASR lattice contains more relaxed word confusions.

#### Semantic and syntactic–acoustic analogy task

We construct an analogy question test set by substituting the words in the original analogy question test set from Mikolov et al. (2013a) with their respective homophones. Considering all the five types of semantic questions and nine types of syntactic questions, for any words in the analogies with homophone alternatives, we swap with the homophone. We prune all the original analogy questions having no words with homophone alternatives. For analogies having more than one words with homophone alternatives, we list all permutations. We found that the number of questions generating by the above method, being exhaustive, was large and hence we randomly sample from the list to retain 948 semantic questions and 6,586 syntactic questions. Table 2 lists a few examples with single and multiple homophone substitutions for semantic and syntactic–acoustic analogy task from our data set.

#### Acoustic similarity task

To create a set of word pairs scored by their acoustic similarity, we add all the homophone word pairs with an acoustic similarity score of 1.0. To get a more diverse range of acoustic similarity scores, we also utilize all the 353 word pairs from the WordSim-353 dataset and compute the normalized phone edit distance using the CMU pronunciation dictionary (Weide, 1998). The normalized phone edit distance is of the range between 0 and 1. The edit distance of 1 refers to the word pair having almost 0 overlap between their respective phonetic transcriptions and thus being completely acoustically dissimilar and vice-versa. We use 1 – “phone_edit_distance” as the acoustic similarity score between the word pair. Thus a score of 1.0 signifies that the two words are identically sounding, whereas a score of 0 refers to words sounding drastically dissimilar. In the case of a word having more than one phonetic transcriptions (pronunciation alternatives), we use the minimum normalized edit distance. Table 3 shows a few randomly picked examples from the generated dataset.

Finally, for evaluation, the respective corpora are pruned to match the in-domain training dataset vocabulary. Table 4 lists the samples in each evaluation dataset before and after pruning.

**Table 4:** Statistics of evaluation datasets.

Task	Total samples	Retained samples
Semantic&Syntactic analogy	19,544	11,409
Acoustic analogy	20,000	2,678
Semantic&Syntactic–acoustic analogy	7,534	3,860
WordSim-353	353	330
Acoustic confusion ratings	1,372	943

### Performance evaluation criterion

In the original work by Mikolov et al. (2013a), the efficiency of the vector representation is measured using the accuracy achieved on the analogy test set. But, in our case, note that the semantic and syntactic analogy task and the semantic and syntactic–acoustic analogy task are mutually exclusive of each other. In other words, the model can get only one, either one, of the analogies correct, meaning any increments with one task will result in decrements over the other task. Moreover, while jointly modeling two orthogonal information streams (i) contextual co-occurrences, and (ii) acoustic word confusions, finding the nearest word vector nearest to the specific analogy is no longer an optimal evaluation strategy. This is because the word vector nearest to the analogy operation can either be along the contextual axis or the confusion axis, that is, each analogy could possibly have two correct answers. For example, the analogy “write”–“wrote”: “read” can be right when the nearest word vector is either “read” (contextual dimension) or “red” (confusion dimension). To incorporate this, we provide the accuracy over top-2 nearest vectors, that is, we count the analogy question as correct if any of the top-2 nearest vectors satisfies the analogy. This also holds for the acoustic confusion analogy tasks, especially for relations involving triplet homophones. For example, the analogy “write”−“right”: “road” can be right when the nearest word vector is either “rode” or “rowed” (for triplet homophones “road”/“rode”/“rowed”). Thus, we present evaluations by comparing the top-1 (nearest vector) evaluation with baseline word2vec against the top-2 evaluation for the proposed confusion2vec models. To maintain consistency, we also provide the top-2 evaluations for the baseline word2vec models in the appendix.

Moreover, since we have three different analogy tasks, we provide the average accuracy among the three tasks in order to have an easy assessment of the performance of various proposed models.

## Results

Table 5 lists the results for various models. We provide evaluations on three different analogy tasks and two similarity tasks as discussed in the section “Evaluation Methods.” Further, more thorough results with the semantic and syntactic accuracy splits are provided under the appendix to gain deeper insights.

**Table 5:** Results: different proposed models.

Model	Analogy tasks				Similarity tasks	
	S&S (%)	Acoustic (%)	S&S–acoustic (%)	Average accuracy (%)	Word similarity	Acoustic similarity
Google W2V	**61.42**	0.9	16.99	26.44	**0.6893**	−0.3489
In-domain W2V	35.15	0.3	7.86	14.44	0.5794	−0.2444
C2V-1	43.33	1.16	15.05	19.85	0.4992	0.1944
C2V-a	22.03	52.58	14.61	29.74	0.105*	**0.8138**
C2V-c	36.15	**60.57**	20.44	**39.05**	0.2937	0.8055
C2V-*	30.53	53.55	**29.35**	37.81	0.0963*	0.7858

**Notes:**

C2V-1, top-confusion; C2V-a, intra-confusion; C2V-c, inter-confusion; C2V-*, hybrid intra-inter; S&S, Semantic & Syntactic analogy.

All the models are of 256 dimensions except Google’s W2V which is 300 dimensions. For the analogy tasks: the accuracies of baseline word2vec models are for top-1 evaluations, whereas of the other models are for top-2 evaluations (as discussed in the section “Analogy Tasks”). Detailed semantic analogy and syntactic analogy accuracies, the top-1 evaluations and top-2 evaluations for all the models are available under Appendix in Table A1. For the similarity tasks: all the correlations (Spearman’s) are statistically significant with *p* < 0.001 except the ones with the asterisks. Detailed *p*-values for the correlations are presented under Appendix in Table A2.

Bold entries correspond to the best results in their respective tasks.

### Baseline Word2Vec model

We consider two variations of Word2Vec baseline model. First, we provide results with the Google’s Word2Vec model (https://code.google.com/archive/p/word2vec) which is trained with orders more training data, and is thus a high upper bound on the semantic and syntactic task. The Google’s Word2Vec model was pruned to match the vocabulary of our corpora to make the evaluation comparable. Second, we consider the Word2Vec model trained on the in-domain ground truth transcripts. The two baseline models result in good performance on Semantic&Syntactic analogy tasks and word similarity task as expected. The Google’s model achieves an accuracy of 61.42% on the Semantic&Syntactic analogy task. We note that the Syntactic accuracy (70.79%) is much higher than the Semantic accuracy (28.98%) (see Table A1). This could be due to our pruned evaluation test set (see Table 4). The in-domain model improves on the Semantic accuracy while losing on the syntactic accuracy over the Google model (see Table A1). The shortcomings of the in-domain model compared to the Google Word2Vec on the Semantic&Syntactic analogy task can be attributed toward the amount of training data and its extensive vocabulary. The in-domain model is trained on 20.8 million words vs. the 100 billion of Google’s News dataset. Moreover, the vocabulary size of in-domain models is approximately 42,150 vs. the three million of Google (Mikolov et al., 2013c) and thus unfair to compare with the rest of the models. Further, evaluating the Acoustic analogy and Semantic&Syntactic–Acoustic analogy tasks, all the baseline models perform poorly. An unusual thing we note is that the Google Word2Vec model performs better comparatively to the in-domain baseline model in the Semantic&Syntactic–Acoustic analogy task. A deeper examination revealed that the model compensates well for homophone substitutions on Semantic&Syntactic analogies which have very similar spellings. This suggests that the typographical errors present in the training data of the Google model results in a small peak in performance for the Semantic&Syntactic–Acoustic analogy task. On the evaluations of similarity tasks, all the baseline models perform well on the word similarity tasks as expected. However, they exhibit poor results on the acoustic similarity task. Overall, the results indicate that the baseline models are largely inept of capturing any relationships over the acoustic word confusions present in a confusion network or a lattice. In our specific case, the baseline models are poor in capturing relationships between acoustically similar words.

### Top-confusion—C2V-1

Comparing the top-confusion (C2V-1 for short), training scheme with the baseline in-domain word2vec model, we observe the baseline model trained on clean data performs better on the Semantic&Syntactic analogy task as expected. Baseline in-domain word2vec achieves 35.15% on the Semantic&Syntactic analogy task whereas the top-confusion model achieves 34.27% (see Table A1). However, the performance difference is minimal. This is encouraging because the top-confusion model is trained on the noisy ASR transcripts. Moreover, we see the noisy transcripts negatively affect the semantic accuracies while the syntactic accuracy remains identical which makes sense (see Table A1). Similar to the baseline in-domain word2vec model, the top-confusion model falls short to Google word2vec mainly due to the extensive amount of data employed in the latter case.

Evaluating for Acoustic analogies and Semantic&Syntactic–Acoustic analogies, the top-confusion scheme improves slightly over the baseline word2vec model. This hints at the ability of the top-confusion model to capture some acoustic word confusions through context (e.g., “take a seat” is expected but sometimes we may see “take a sit”). The improvements are small because in a good quality ASR the top confusion network hypotheses contain few errors thus context learning is much stronger and acoustic-confusion learning is minimal. Note that the top-confusion model would converge to the baseline word2vec model in the case of a zero word error rate.

Further, inspecting the performance in the similarity task, the top-confusion model exhibits statistically significant positive correlation in the word similarity task, although slightly smaller correlation than the baseline word2vec and Google word2vec model. However, we observe a positive (statistically significant) correlation on the acoustic similarity task, whereas both the baseline word2vec and Google word2vec model exhibit a negative correlation. This further validates the proposed top-confusion model’s capability to capture acoustic word confusions.

### Intra-confusion, C2V-a

With intra-confusion training (C2V-acoustic or C2V-a for short) we expect the model to capture acoustically similar word relationships, while completely ignoring any contextual relations. Hence, we expect the model to perform well on acoustic analogies and acoustic similarity tasks and to perform poorly on Semantic&Syntactic analogies and word similarity tasks. The Table 5 lists the results obtained using intra-confusion training. The results are in conjunction with our expectations. The model gives the worst results in Semantic&Syntactic analogy task. However, we observe that the syntactic analogy accuracy to be a fair amount higher than the semantic accuracy (see Table A1). We think this is mainly because of syntactically similar words appearing along the word confusion dimension in the confusion networks, resultant of the constraints enforced on the confusion network by the (ASR) language model—which are known to perform better for syntactic tasks (Mikolov et al., 2013a). The model also gives the highest correlation on the acoustic similarity task, while performing poorly on the word similarity task.

### Inter-confusion, C2V-c

With inter-confusion training (C2V-contextual or C2V-c for short), we hypothesized that the model is capable of jointly modeling both the contextual information as well as confusions appearing contextually. Hence, we expect the model to perform well on both the Semantic&Syntactic analogy and Acoustic analogy tasks and in doing so result in better performance with Semantic&Syntactic–Acoustic analogy task. We also expect the model to give high correlations for both word similarity and acoustic similarity tasks. From Table 5, we observe that as hypothesized the inter-confusion training shows improvements in the Semantic&Syntactic analogy task. Quite surprisingly, the inter-confusion training shows better performance than the intra-confusion training for the Acoustic analogy task, hinting that having good contextual representation could mutually be beneficial for the confusion representation. However, we don’t observe any improvements in the Semantic&Syntactic–Acoustic analogy task. Evaluating on the similarity tasks, the results support the observations drawn from analogy tasks, that is, the model fares relatively well in both word similarity and acoustic similarity.

### Hybrid intra–inter confusion, C2V-*

The hybrid intra–inter confusion training (C2V-* for short) introduces all confusability and allows learning directly confusable acoustic terms, such as in the C2V-a case, and contextual information that incorporates confusable terms, as in the Inter or C2V-c case. This model shows comparable performance in jointly modeling on both the Semantic&Syntactic and Acoustic analogy tasks. One crucial observation is that it gives significantly better performance with the Semantic&Syntactic–Acoustic analogy task. This suggests that jointly modeling both the intra-confusion and inter-confusion word mappings is useful. However, it achieves better results by compromising on the semantic analogy (see Table A1) accuracy and hence also negatively affecting the word similarity task. The model achieves good correlation on the acoustic similarity task.

Overall, our proposed Confusion2Vec models capture significantly more useful information compared to the baseline models judging by the average accuracy over the analogy tasks. One particular observation we see across all the proposed models is that the performance remains fairly poor for the Semantic&Syntactic–Acoustic analogy task. This suggests that the Semantic&Syntactic–Acoustic analogy task is inherently hard to solve. We believe that to achieve better results with Semantic&Syntactic–Acoustic analogies, it is necessary to have a robust performance on one of the tasks (Semantic&Syntactic analogies or Acoustic analogies) to begin with, that is, better model initialization could help. Next, we experiment with model initializations/pre-training.

### Model initialization/pre-training

Table 6 lists the results with model initialization/pre-training. The in-domain word2vec baseline model and the top-confusion models are initialized from the Google Word2Vec model. Pre-training the models provide improvements with Semantic&Syntactic analogy results to be close and comparable to that of the Google’s Word2Vec model. Empirically, we find the top-confusion model inherits approximately similar contextual information as the baseline models, and in addition outperforms the baseline in average accuracy. Thus, for future experiments we adopt the top-confusion model (rather than word2vec model) for initialization, model concatenation, and joint-training. The remaining models (C2V-a, C2V-c, and C2V-*) are initialized from the top-confusion model (i.e., C2V-1, the top-confusion model initialized from Google Word2Vec), since this would enable full compatibility with the vocabulary. Since the Google Word2Vec model is 300 dimensional, this forces all the pre-trained models (Table 6) to be 300, as opposed to 256 dimensions (Table 5).

**Table 6:** Results with pre-training/initialization.

Model	Analogy tasks				Similarity tasks	
	S&S (%)	Acoustic (%)	S&S–acoustic (%)	Average accuracy (%)	Word similarity	Acoustic similarity
Google W2V	**61.42**	0.9	16.99	26.44	**0.6893**	−0.3489
In-domain W2V	59.17	0.6	8.15	22.64	0.4417	−0.4377
C2V-1	61.13	0.9	16.66	26.23	**0.6036**	−0.4327
C2V-a	63.97	16.92	**43.34**	41.41	0.5228	0.62
C2V-c	**65.45**	**27.33**	38.29	**43.69**	0.5798	0.5825
C2V-*	65.19	20.35	42.18	42.57	0.5341	**0.6237**

**Notes:**

C2V-1, top-confusion; C2V-a, intra-confusion; C2V-c, inter-confusion; C2V-*, hybrid intra-inter; S&S, Semantic & Syntactic Analogy.

All the models are of 300 dimensions. For the analogy tasks: the accuracies of baseline word2vec models are for top-1 evaluations, whereas of the other models are for top-2 evaluations (as discussed in the section “Analogy Tasks”). Detailed semantic analogy and syntactic analogy accuracies, the top-1 evaluations and top-2 evaluations for all the models are available under Appendix in Table A3. For the similarity tasks: all the correlations (Spearman’s) are statistically significant. Detailed *p*-values for the correlations are presented under Appendix in Table A4.

Bold entries correspond to the best results in their respective tasks.

For intra-confusion model, the pre-training provides drastic improvements on Semantic&Syntactic analogy task at the expense of the Acoustic analogy task. Even-though the accuracy of Acoustic analogy task decreases comparatively to without pre-training, it remains significantly better than the baseline model. More importantly, the Semantic&Syntactic–Acoustic analogy task accuracy doubles. Inter-confusion model does not compromise on the Semantic&Syntactic analogy tasks, in doing so gives comparable performances to the baseline model. Additionally, it also does well on the Acoustic and Semantic&Syntactic–Acoustic analogy task as was the case without pre-training. In the case of hybrid intra-inter confusion model, similar trends are observed as was with no pre-training, but with considerable improvements in accuracies. Pre-training also helps in boosting the correlations for the word similarity tasks for all the models. Overall, we find the pre-training to be extremely useful.

### Model concatenation

Table 7 (rows 3-5) lists the results with model concatenation. We concatenate each of the proposed models (Table 5) with the pre-trained top-confusion model (we use C2V-1 model instead of word2vec as hypothesized in Fig. 6B because empirically C2V-1 model provided similar performance on Semantic&Syntactic tasks and overall better average accuracy on analogy tasks compared to the baseline-in-domain W2V model). Thus the resulting vector space is 556 dimensional (300 (pre-trained top-confusion model) + 256 (proposed models from Table 5)). In our case, we believe the dimension expansion of the vector space is insignificant in terms of performance considering the relatively low amount of training data compared to Google’s word2vec model. To be completely fair in judgment, we create a new baseline model with 556 dimensional embedding space for comparison. To train the new baseline model, the 556 dimension embedding was initialized with 300 dimensional Google’s word2vec embedding and the rest of the dimensions as zeros (null space). Comparing the 556 dimension baseline from Table 7 with the previous 300 dimensional baseline from Table 6, the results are almost identical which confirms the dimension expansion is insignificant with respect to performance.

**Table 7:** Model concatenation and joint optimization results.

Model	Fine-tuning scheme	Analogy tasks				Similarity tasks	
		S&S	Acoustic	S&S–acoustic	Average	Word	Acoustic
Google W2V	–	61.42%	0.9%	16.99%	26.44%	**0.6893**	−0.3489
In-domain W2V (556 dim.)	–	63.6%	0.81%	14.54%	26.32%	0.6333	−0.4717
**Model concatenation**							
C2V-1 (F) + C2V-a (F)	–	67.03%	25.43%	40.36%	44.27%	0.5102	0.7231
C2V-1 (F) + inter-confusion (F)	–	70.84%	35.25%	35.18%	47.09%	0.5609	0.6345
C2V-1 (F) + hybrid intra-inter (F)	–	68.08%	11.39%	41.3%	40.26%	0.4142	0.5285
**Fixed contextual subspace joint optimization**							
C2V-1 (F) + C2V-a (L)	Inter	71.65%	20.54%	33.76%	41.98%	0.5676	0.4437
C2V-1 (F) + C2V-a (L)	Intra	67.37%	28.64%	39.09%	45.03%	0.5211	0.6967
C2V-1 (F) + C2V-a (L)	Hybrid	70.02%	25.84%	37.18%	44.35%	0.5384	0.6287
C2V-1 (F) + C2V-c (L)	Inter	72.01%	35.25%	33.58%	46.95%	0.5266	0.5818
C2V-1 (F) + C2V-c (L)	Intra	69.7%	39.32%	39.07%	49.36%	0.5156	0.7021
C2V-1 (F) + C2V-c (L)	Hybrid	**72.38%**	37.75%	37.95%	49.36%	0.5220	0.6674
C2V-1 (F) + C2V-* (L)	Inter	71.36%	8.55%	33.21%	37.71%	0.5587	0.302
C2V-1 (F) + C2V-* (L)	Intra	66.85%	13.33%	40.1%	40.09%	0.4996	0.5691
C2V-1 (F) + C2V-* (L)	Hybrid	68.32%	11.61%	38.19%	39.37%	0.5254	0.4945
**Unrestricted joint optimization**							
C2V-1 (L) + C2V-a (L)	Inter	62.12%	46.42%	36.4%	48.31%	0.5513	0.7926
C2V-1 (L) + C2V-a (L)	Intra	64.85%	40.55%	42.38%	49.26%	0.5033	0.7949
C2V-1 (L) + C2V-a (L)	Hybrid	31.65%	61.91%	23.55%	39.04%	0.1067*	**0.8309**
C2V-1 (L) + C2V-c (L)	inter	64.98%	52.99%	34.79%	50.92%	0.5763	0.7725
C2V-1 (L) + C2V-c (L)	Intra	65.88%	49.4%	41.51%	**52.26%**	0.5379	0.7717
C2V-1 (L) + C2V-c (L)	Hybrid	37.86%	**67.21%**	25.96%	43.68%	0.2295	0.8294
C2V-1 (L) + C2V-* (L)	Inter	65.54%	27.97%	36.87%	43.46%	0.5338	0.6953
C2V-1 (L) + C2V-* (L)	Intra	64.42%	20.05%	**42.56%**	42.34%	0.4920	0.6942
C2V-1 (L) + C2V-* (L)	Hybrid	65.79%	22.63%	41.3%	43.24%	0.4967	0.6986

**Notes:**

C2V-1, top-confusion; C2V-a, intra-confusion; C2V-c, inter-confusion; C2V-*, hybrid intra-inter.

All the models are of 556 (300 + 256) dimensions. Acronyms: (F):Fixed embedding, (L):Learn embedding during joint training, S&S: Semantic & Syntactic analogy. For the analogy tasks: the accuracies of baseline word2vec models are for top-1 evaluations, whereas of the other models are for top-2 evaluations (as discussed in the section “Analogy Tasks”). Detailed semantic analogy and syntactic analogy accuracies, the top-1 evaluations and top-2 evaluations for all the models are available under Appendix in Table A5. For the similarity tasks: all the correlations (Spearman’s) are statistically significant with *p* < 0.001 except the ones with the asterisks. Detailed *p*-values for the correlations are presented under Appendix in Table A6.

Bold entries correspond to the best results in their respective tasks.

With model concatenation, we see slightly better results (average analogy accuracy) comparing with the pre-trained models from Table 6, an absolute increase of up-to approximately 5% among the best results. The correlations with similarity tasks are similar and comparable with the earlier results with the pre-trained models.

### Joint optimization

#### Fixed contextual subspace

Rows 6–14 of Table 7 display the results of joint optimization with concatenated, fixed top-confusion (C2V-1) embeddings and learn-able confusion2vec (C2V-a/c/*) embeddings. As hypothesized with fixed subspace, the results indicate better accuracies for the Semantic&Syntactic analogy task. Thereby, the improvements also reflect on the overall average accuracy of the analogy tasks. This also confirms the need for joint optimization which boosts the average accuracy up-to approximately 2% absolute over the unoptimized concatenated model.

#### Unrestricted optimization

The last nine rows of Table 7 display the results obtained by jointly optimizing the concatenated models without constraints. Both the subspaces are fine tuned to convergence with various proposed training criteria. We consistently observe improvements with the unrestricted optimization over the unoptimized model concatenations. In terms of average accuracy we observe an increase in average accuracy by up-to 5% (absolute) approximate over the unoptimized concatenated models. Moreover, we obtain improvements over the fixed contextual subspace joint-optimized models, up-to 2–3% (absolute) in average accuracies. The best overall model in terms of average accuracies is obtained by unrestricted joint optimization on the concatenated top-confusion and inter-confusion models by fine-tuning with the intra-confusion training scheme.

### Results summary

Firstly, comparing among the different training schemes (see Table 5), the inter-confusion training consistently gives the best Acoustic analogy accuracies, whereas the hybrid training scheme often gives the best Semantic&Syntactic–Acoustic analogy accuracies. As far as the Semantic&Syntactic analogy task is concerned, the intra-confusion is often found to give preference to syntactic relations, while the inter-confusion boosts the semantic relations and the hybrid scheme balances both relations (see Table A1). Next, pre-training/initializing the model gives drastic improvements in overall average accuracy of analogy tasks. Concatenating the top-confusion model with the confusion2vec (C2V-a/c/*) model gives slightly better results. More optimizations and fine-tuning over the concatenated model gives considerably the best results.

Overall, the best results are obtained with unrestricted joint optimization of top-confusion and inter-confusion model, that is, fine-tuning using intra-confusion training mode. In terms of average analogy accuracies the confusion2vec model (C2V-a/c/*) outperforms the baseline by up-to 26.06%. The best performing confusion2vec model outperforms the word2vec model even on the Semantic&Syntactic analogy tasks (by a relative 7.8%). Moreover, even the comparison between the top-2 evaluations of both the word2vec and confusion2vec (C2V-1/a/c/*) suggests very similar performance on Semantic&Syntactic-analogy tasks (see Table A5). This confirms and emphasizes that the confusion2vec (C2V-1/a/c/*) doesn’t compromise on the information captured by word2vec but succeeds in augmenting the space with word confusions. Another highlight observation is that modeling the word confusions boost the semantic and syntactic scores of the Semantic&Syntactic analogy task (compared to word2vec), suggesting inherent information in word confusions which could be exploited for better semantic-syntactic word relation modeling.

## Vector Space Analysis

In this section, we compare the vector space plots of the typical word2vec space and the proposed confusion2vec vector space for a specifically chosen set of words. We choose a subset of words representing three categories to reflect semantic relationships, syntactic relationships and acoustic relationships. The vector space representations of the words are then subjected to dimension reduction using principle component analysis (PCA) to obtain 2D vectors which are used for plotting.

### Semantic relationships

**Figure 7:**
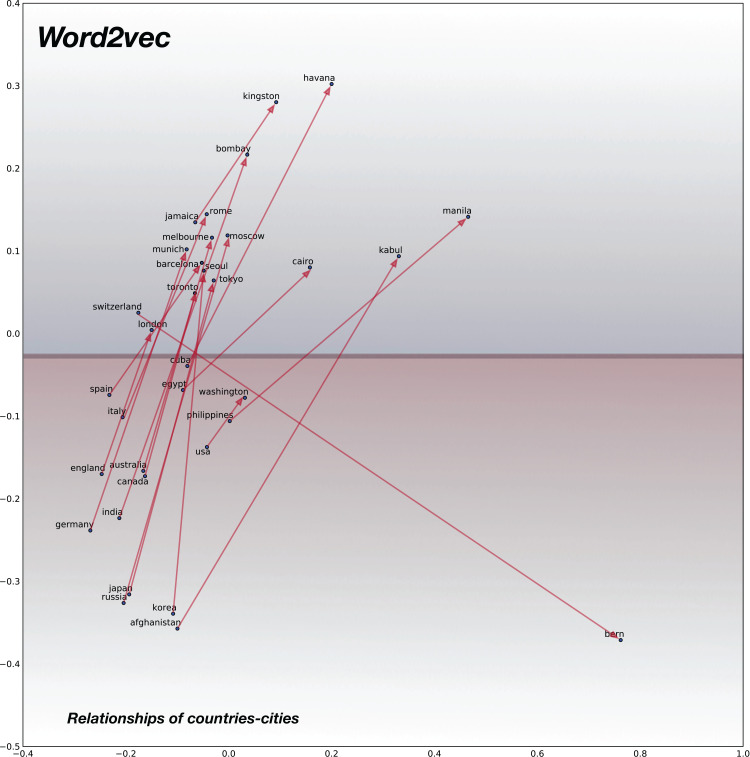
2D plot after PCA of word vector representation on baseline pre-trained word2vecF. Demonstration of semantic relationship on randomly chosen pairs of countries and cities. Country-city vectors are almost parallel/similar. Countries are clustered together on the bottom half (highlighted with red hue) and the cities on upper half (highlighted with blue hue).

**Figure 8:**
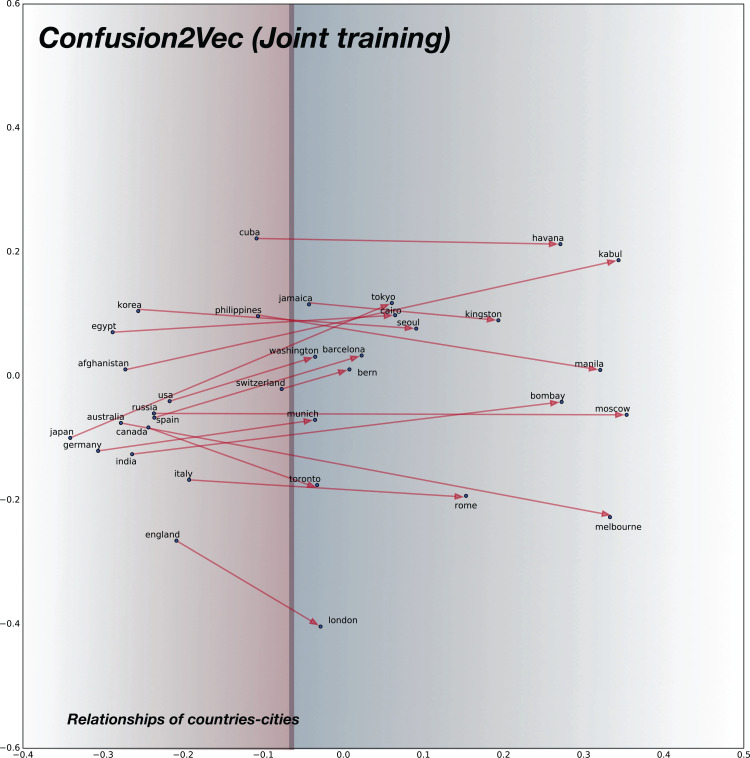
2D plot after PCA of word vector representation on jointly optimized pre-trained C2V-1 + C2V-a models. Demonstration of semantic relationship on randomly chosen pairs of countries and cities. Observe the semantic relationships are preserved as in the case of word2vec model: country-city vectors are almost parallel/similar. Countries are clustered together on the left half (highlighted with red hue) and the cities on right half (highlighted with blue hue).

For analyzing the semantic relationships, we compile random word pairs (constrained by the availability of these in our training data) representing Country–Cities relationships. The 2D plot for baseline pre-trained word2vec model is shown in Fig. 7 and for the proposed confusion2vec model, specifically for the randomly selected, jointly-optimized top-confusion + intra-confusion model (corresponding to row 7 in Table 7) is displayed in Fig. 8. The following observations can be made comparing the two PCA plots:

- Examining the baseline word2vec model, we find the Cities are clustered over the upper half of the plot (highlighted with blue hue in Fig. 7) and Countries are clustered together at the bottom half (highlighted with red hue in Fig. 7).
- Similar trends are observed with the proposed confusion2vec model, where the cities are clustered together over the right half of the plot (highlighted with blue hue in Fig. 8) and the countries are grouped together toward the left half (highlighted with red hue in Fig. 8).
- In the Word2Vec space, the vectors of Country–City word pairs are roughly parallel, pointing north-east (i.e., vectors are approximately similar).
- Similar to the word2vec space, with the Confusion2Vec, we observe the vectors of Country–City word pairs are fairly parallel and point to the east (i.e., vectors are highly similar).
  The four observations indicate that the Confusion2Vec preserves the Semantic relationships between the words (similar to the Word2Vec space).

### Syntactic relationships

**Figure 9:**
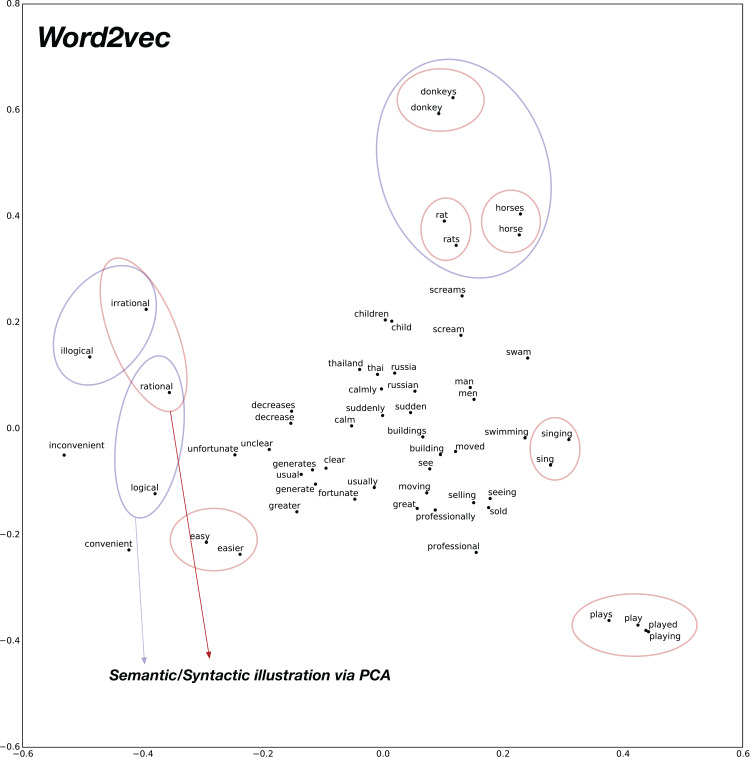
2D plot after PCA of word vector representation on baseline pre-trained word2vec. Demonstration of syntactic relationship on randomly chosen 30 pairs of adjective-adverb, opposites, comparative, superlative, present-participle, past-tense, plurals. Observe the clustering of syntactically related words (Ex: highlighted with red ellipses). Few semantically related words are highlighted with blue ellipses (Ex: animals).

**Figure 10:**
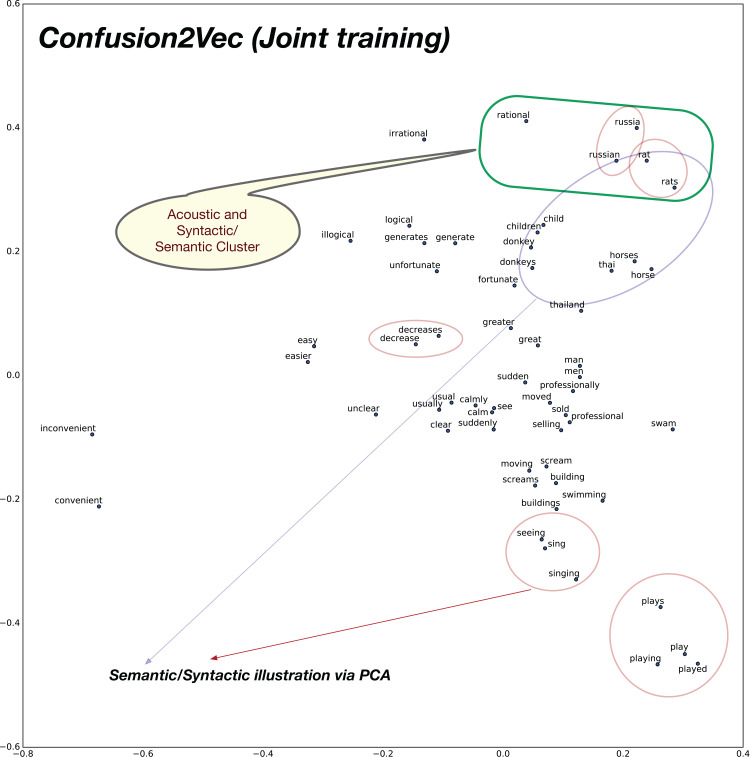
2D plot after PCA of word vector representation on jointly optimized pre-trained C2V-1 + C2V-a models. Demonstration of syntactic relationship on randomly chosen 30 pairs of adjective-adverb, opposites, comparative, superlative, present-participle, past-tense, plurals. Syntactic clustering is preserved by confusion2vec similar to word2vec. Red ellipses highlight few examples of syntactically related words. Similar to word2vec, semantically related words (Ex: animals), highlighted with blue ellipses, are also clustered together. Additionally confusion2vec clusters acoustically similar words together (indicated with green ellipse).

To analyze the syntactic relationships, we create 30 pairs of words composed of Adjective-Adverb, Opposites, Comparative, Superlative, Present-Participle, Past-tense, Plurals. The PCA 2D plots for baseline pre-trained word2vec model and the proposed confusion2vec model are illustrated in Figs. 9 and 10, respectively. The following inferences can be made from the two plots:

- Inspecting the baseline word2vec model, we observe that the word pairs depicting syntactic relations occur often close-by (highlighted with red ellipses in Fig. 9).
- Few semantic relations are also apparent and are highlighted with blue ellipses in Fig. 9. For example, animals are clustered together.
- Similarly, with the Confusion2Vec model, we observe syntactic clusters of words highlighted with red ellipses in Fig. 10.
- Semantic relations apparent in the case of word2vec is also evident with the Confusion2Vec, which are highlighted with blue ellipses in Fig. 10.
- Additionally, with the Confusion2Vec model, we find clusters of acoustically similar words (with similar phonetic transcriptions). These are highlighted using a green ellipse in Fig. 10.

The above findings confirm that the confusion2vec models preserve the syntactic relationships similar to word2vec models, supporting our hypothesis.

### Acoustic relationships

**Figure 11:**
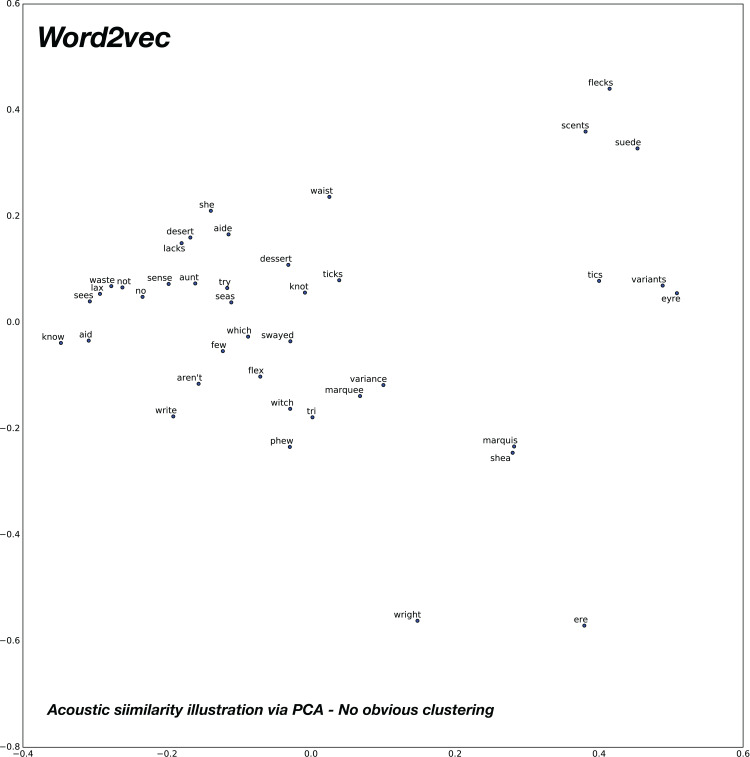
2D plot after PCA of word vector representation on baseline pre-trained word2vec. Demonstration of vector relationship on randomly chosen 20 pairs of acoustically similar sounding words. No apparent relations between acoustically similar words are evident.

**Figure 12:**
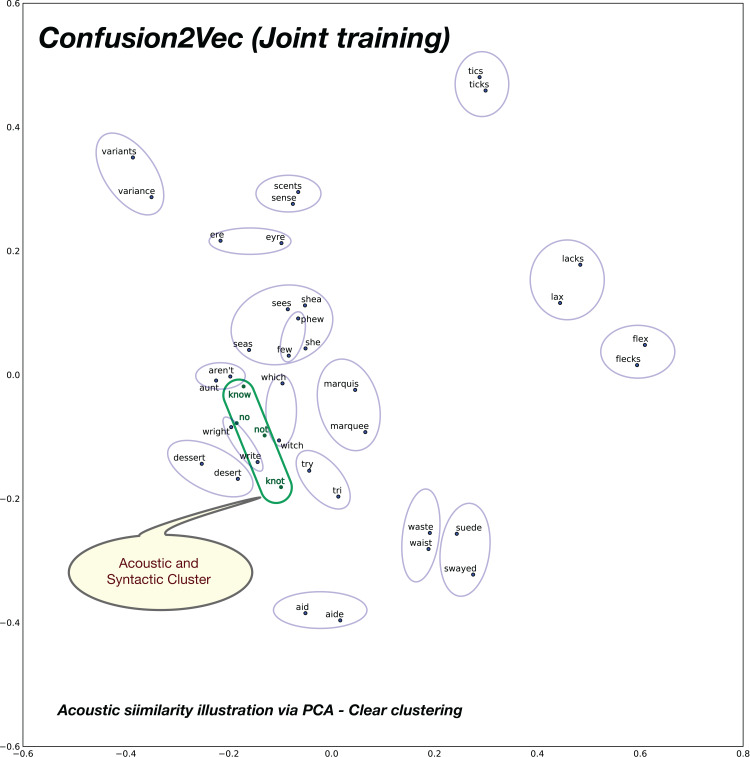
2D plot after PCA of word vector representation on jointly optimized pre-trained C2V-1 + C2V-a models. Demonstration of vector relationship on randomly chosen 20 pairs of acoustically similar sounding words. Confusion2Vec clusters acoustically similar words together (highlighted with blue ellipses). Additionally, inter-relations between syntactically related words and acoustically related words are also evident (highlighted with a green ellipse).

In order to analyze the relationships of similar sounding words in the word vector spaces under consideration, we compose 20 pairs of acoustically similar sounding words, with similar phonetic transcriptions. The 2D plot obtained after PCA for the baseline word2vec model is shown in Fig. 11 and the proposed confusion2vec model is shown in Fig. 12. We make the following observations from the two figures:

- Observing the baseline Word2vec model, no apparent trends are found between the acoustically similar words. For example, there is no trivial relationships apparent from the plot in Fig. 11 between the word “no” and “know,” “try” and “tri,” etc.
- However, inspecting the proposed confusion2vec model, there is an obvious trend apparent, the acoustically similar words are grouped together in pairs and occur roughly in similar distances. The word pairs are highlighted with blue ellipses in Fig. 12.
- Additionally, in Fig. 12, as highlighted in a green ellipse, we observe the four words “no,” “not,” “knot,” and “know” occur in close proximity. The word pair “no” and “not” portray semantic/syntactic relation whereas the pairs “knot” & “not” and “no” & “know” are acoustically related.

The above findings suggest that the word2vec baseline model fails to capture any acoustic relationships whereas the proposed confusion2vec successfully models the confusions present in the lattices, in our specific case the acoustic confusions from the ASR lattices.

## Discussion

**Figure 13:**
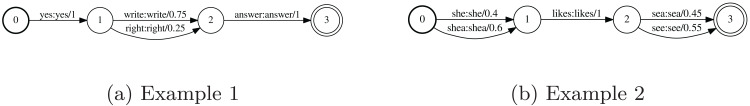
Confusion network examples.

**Figure 14:**
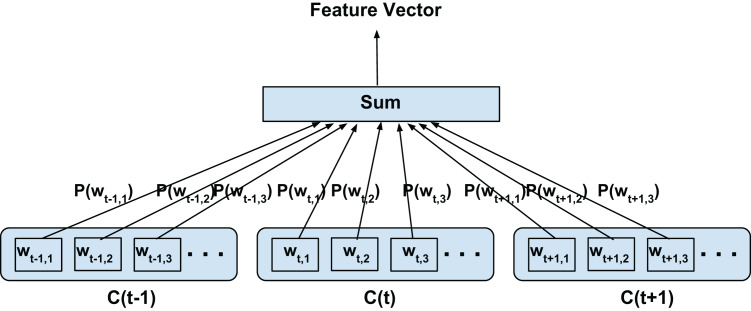
Computation of lattice feature vector.

In this section, we demonstrate why the proposed embedding space is superior for modeling word lattices with the support of toy examples. Let’s consider a simple task of ASR error correction. As shown by Allauzen (2007), Ogata & Goto (2005) and Shivakumar et al. (2018), often, the information needed to correct the errors are embedded in the lattices. The toy examples in Figs. 13A and 13B depict the real scenarios encountered in ASR. The lattice feature representation is a weighted vector sum of all words in the confusion and its context present in the lattice (see Fig. 14). We compare the proposed confusion2vec embeddings with the popular word2vec using cosine similarity as the evaluation measure. Table 8 lists the evaluation for the following cases: (i) ASR output is correct, (ii) ASR output is wrong and the correct candidate is present in the lattice, (iii) ASR output is wrong and the correct candidate is absent from the lattice, and (iv) ASR output is wrong and with no lattice available. The following observations are drawn from the results:

1. Confusion2vec shows higher similarity with the correct answers when the ASR output is correct (see Table 8; Example 1.1, 2.1).
2. Confusion2vec exhibits higher similarity with the correct answers when the ASR output is wrong—meaning the representation is closer to the correct candidate and therefore more likely to correct the errors (see Table 8; Example 1.2, 2.2, 1.3, 2.3).
3. Confusion2vec yields high similarity even when the correct word candidate is not present in the lattice—meaning confusion2vec leverages inherent word representation knowledge to aid re-introduction of pruned or unseen words during error correction (see Table 8; Example 1.4, 1.5, 1.6).
4. The confusion2vec shows low similarity in the case of fake lattices with highly unlikely word alternatives (see Table 8; Example 2.4, 2.5).

**Table 8:** Cosine similarity between the ASR ground-truth and ASR output in application to ASR error correction for baseline pre-trained word2vec and the proposed confusion2vec: jointly optimized intra-confusion + top-confusion models.

Example	Ground-truth	ASR output	W2V similarity	C2V similarity
1.1	“Yes right answer”	“Yes (right/write) answer”	0.96190	0.96218
1.2	“Yes right answer”	“Yes write answer”	0.93122	0.93194
1.3	“Yes write answer”	“Yes (right/write) answer”	0.99538	0.99548
1.4	“Yes rite answer”	“Yes (right/write) answer”	0.84216	0.88206
1.5	“Yes rite answer”	“Yes right answer”	0.86003	0.87085
1.6	“Yes rite answer”	“Yes write answer”	0.82073	0.87034
2.1	“She likes sea”	“(She/shea) likes (see/sea)”	0.91086	0.92130
2.2	“She likes sea”	“Shea likes see”	0.73295	0.77137
2.3	“Shea likes see”	“(She/shea) likes (see/sea)”	0.94807	0.95787
2.4	“Shea likes see”	“(She/shea) likes (see/rocket)”	0.93560	0.93080
2.5	“She likes sea”	“(She/shea) likes (see/rocket)”	0.85853	0.85757

**Note:**

Examples 1.1–1.6 inherits structure as in Fig. 13A, that is, “yes (right/write) answer” assigns weight of 1.0 to yes and answer, 0.75 to right, 0.25 to write. Similarly Examples 2.1–2.5 inherits structure as in Fig. 13B.

All the above observations are supportive of the proposed confusion2vec word representation and is in line with the expectations for the task of ASR error correction.

## Potential Applications

In addition to the above discussed ASR error correction task, other potential applications include:

**Machine Translation:** In Machine Translation, word lattices are used to provide multiple sources for generating a single translation (Schroeder, Cohn & Koehn, 2009; Dyer, 2010). Word lattices derived from reordered hypotheses (Costa-Jussà & Fonollosa, 2007; Niehues & Kolss, 2009; Hardmeier, Bisazza & Federico, 2010), morphological transformations (Dyer, 2007; Hardmeier, Bisazza & Federico, 2010), word segmentations (Dyer, 2009), paraphrases (Onishi, Utiyama & Sumita, 2011) are used to introduce ambiguity and alternatives for training machine translation systems (Wuebker & Ney, 2012; Dyer, Muresan & Resnik, 2008; Dyer, 2010). Source language alternatives can also be exploited by introducing ambiguity derived from the combination of multiple machine translation systems (Matusov, Ueffing & Ney, 2006; Rosti et al., 2007a; Rosti, Matsoukas & Schwartz, 2007b). In the case of Machine Translation, the word-confusion subspace is associated with morphological transformations, word segmentations, paraphrases, part-of-speech information, etc., or a combination of them. Although the word-confusion subspace is not orthogonal, the explicit modeling of such ambiguity relationships is beneficial.

**NLP:** Other NLP based applications like paraphrase generation (Quirk, Brockett & Dolan, 2004), word segmentation (Kruengkrai et al., 2009), part-of-speech tagging (Kruengkrai et al., 2009) also operate on lattices. As discussed in the section “Machine Learning Algorithms,” confusion2vec can exploit the ambiguity present in the lattices for the betterment of the tasks.

**ASR:** In ASR systems, word lattices and confusion networks are often re-scored using various algorithms to improve their performances by exploiting ambiguity (Sundermeyer et al., 2014; Mangu, Brill & Stolcke, 2000; Xiong et al., 2016; Liu et al., 2014). In the case of ASR, the word-confusion subspace is associated with the acoustic similarity of words which is often orthogonal to the semantic-syntactic subspace as discussed in the section “Human Speech Production, Perception and Hearing.” Examples 1–3 are prime cases supporting the need for jointly modeling acoustic word confusions and semantic-syntactic subspace.

**Spoken Language Understanding:** Similarly, as in the case of ASR, Confusion2Vec could exploit the inherent acoustic word-confusion information for keyword spotting (Mangu, Brill & Stolcke, 2000), confidence score estimation (Mangu, Brill & Stolcke, 2000; Seigel & Woodland, 2011; Kemp & Schaaf, 1997; Jiang, 2005), domain adaptation (Shivakumar et al., 2018), lattice compression (Mangu, Brill & Stolcke, 2000), spoken content retrieval (Chelba, Hazen & Saraclar, 2008; Hori et al., 2007), system combinations (Mangu, Brill & Stolcke, 2000; Hoffmeister et al., 2007), and other spoken language understanding tasks (Hakkani-Tür et al., 2006; Tur et al., 2002; Marin et al., 2012) which operate on lattices.

**Speech Translation:** In speech translation systems, incorporating the word lattices and confusion networks (instead of the single top hypothesis) is beneficial in better integrating speech recognition system to the machine translation systems (Bertoldi, Zens & Federico, 2007; Mathias & Byrne, 2006; Matusov, Kanthak & Ney, 2005; Schultz et al., 2004). Similarly, exploiting uncertainty information between the “ASR—Machine Translation—Speech synthesis” systems for speech-to-speech translation is useful (Lavie et al., 1997; Wahlster, 2013). Since speech translation involves combination of ASR and the Machine Translation systems, the word-confusion subspace is associated with a combination of acoustic word-similarity (for ASR) and morphological-segmentation-paraphrases ambiguities (for Machine Translation).

“See son winter is here” → “voir fils hiver est ici”    (Example 4)
“Season winter is here” → “saison hiver est ici”     (Example 5)

Examples 4 and 5 demonstrate a case of speech translation of identically sounding English phrases to French. Words “See son” and “Season” demonstrate ambiguity in terms of word segmentation. Whereas, the phrases “See son” and “Season” also exhibit ambiguity in terms of acoustic similarity. By modeling both word-segmentation and acoustic-confusion through word vector representations, the confusion2vec can provide crucial information that the French words “voir” and “saison” are confusable under speech translation framework.

**Optical Character Recognition:** In optical character recognition (OCR) systems, the confusion axis is related to pictorial structures of the characters/words. For example, say the characters “a” and “o” are easily confusable thus leading to similar character vectors in the embedding space. In the case of word level confusions leading to words “ward” and “word” being similar with confusion2vec (word2vec would have the words “word” and “ward” fairly dissimilar). Having this crucial optical confusion information is useful during OCR decoding on sequence of words when used in conjunction with the linguistic contextual information.

**Image/Video Scene Summarization:** The task of scene summarization involves generating descriptive text summarizing the content in one or more images. Intuitively, the task would benefit from linguistic contextual knowledge during the text generation. However, with the confusion2vec, one can model and expect to capture two additional information streams (i) pictorial confusion of image/object recognizer, and (ii) pictorial context, that is, modeling objects occurring together (e.g., we can expect oven to often appear nearby a stove or other kitchen appliances). The additional streams of valuable information embedded in the lattices can contribute for better decoding. In other words, for example, word2vec can exhibit high dissimilarity between the words “lifebuoy” and “donuts”, however, the confusion2vec can capture their pictorial similarity in a better word space representation and thus aiding in their end application of scene summarization.

## Conclusion

In this work, we proposed a new word vector representation motivated from human speech and perception and aspects of machine learning for incorporating word confusions from lattice like structures. The proposed confusion2vec model is meant to capture additional word-confusion information and improve upon the popular word2vec models without compromising the inherent information captured by the word2vec models. Although the word confusions could be domain/task specific, we present a case study on ASR lattices where the confusions are based on acoustic similarity of words. Specifically, with respect to ASR related applications, the aim is to capture the contextual statistics, as with word2vec, and additionally also capture the acoustic word confusion statistics. Several training configurations are proposed for confusion2vec model, each utilizing different degrees of acoustic confusability vs. contextual information, present in the noisy (confusion network) ASR output, for modeling the word vector space. Further, techniques like pre-training/initializations, model concatenation and joint optimization are proposed and evaluated for the confusion2vec models. Appropriate evaluation schemes are formulated for the domain specific application. The evaluation schemes are inspired from the popular analogy based question test set and word similarity tasks. A new analogy task and word similarity tasks are designed for the acoustic confusion/similarity scenario. A detailed tabulation of results are presented for the confusion2vec model and compared to the baseline word2vec models.

The results show that the confusion2vec can augment additional task-specific word confusion information without compromising on the semantic and syntactic relationships captured by the word2vec models. Next, detailed analysis is conducted on the confusion2vec vector space through PCA reduced two-dimensional plots for three independent word relations: (i) Semantic relations, (ii) Syntactic relations, and (iii) Acoustic relations. The analysis further supports our aforementioned experimental inferences. Few toy examples are presented toward the task of ASR error correction to support the adequacy of the Confusion2vec over the word2vec word representations. The study validates through various hypotheses and test results, the potential benefits of the confusion2vec model.

## Future Work

In the future, we plan to work on improving the confusion2vec model by incorporating the sub-word and phonemic transcription of words during training. Sub-words and character transcription information is shown to improve the word vector representation (Bojanowski et al., 2017; Chen et al., 2015). We believe the sub-words and phoneme transcriptions of words are even more relevant to confusion2vec. In addition to the improvements expected toward the semantic and syntactic representations (word2vec), since the sub-words and phoneme transcriptions of acoustically similar words are similar, it should help in modeling the confusion2vec to a much greater extent.

Apart from concentrating on improving the confusion2vec model, this work opens new possible opportunities in incorporating the confusion2vec embeddings to a whole range of full-fledged applications such as ASR error correction, speech translation tasks, machine translation, discriminative language models, optical character recognition, image/video scene summarization, etc.

## Appendix

**Table A1:** Analogy task results with Semantic&Syntactic splits: different proposed models.

Model	Analogy tasks							
	Semantic&Syntactic analogy			Acoustic analogy	Semantic&Syntactic–acoustic analogy			Average accuracy
	Semantic	Syntactic	Semantic&Syntactic		Semantic–acoustic	Syntactic–acoustic	Semantic&Syntactic–acoustic	
Google W2V	28.98% (35.75%)	70.79% (78.74%)	61.42% (69.1%)	0.9% (1.42%)	6.54% (14.38%)	17.9% (27.46%)	16.99% (26.42%)	26.44% (32.31%)
In-domain W2V	42.39% (51.57%)	33.14% (43.14%)	35.15% (44.98%)	0.3% (0.6%)	5.17% (10.69%)	8.13% (11.93%)	7.86% (11.82%)	14.44% (19.13%)
C2V-1	38.33% (46.7%)	33.1% (42.36%)	34.27% (43.33%)	0.7% (1.16%)	11.76% (14.38%)	11.23% (15.11%)	11.27% (15.05%)	15.41% (19.85%)
C2V-a	0.51% (0.78%)	18.59% (28.17%)	14.54% (22.03%)	41.93% (52.58%)	0.98% (2.29%)	9.62% (15.67%)	8.94% (14.61%)	21.8% (29.74%)
C2V-c	16.15% (23.7%)	26.14% (39.74%)	23.9% (36.15%)	48.58% (60.57%)	3.27% (6.86%)	12.13% (21.61%)	11.42% (20.44%)	27.97% (39.05%)
C2V-*	2.07% (2.58%)	28.91% (38.6%)	22.89% (30.53%)	40.78% (53.55%)	1.96% (2.94%)	20.99% (31.63%)	19.48% (29.35%)	27.72% (37.81%)

**Notes:**

C2V-1, top-confusion; C2V-a, intra-confusion; C2V-c, inter-confusion; C2V-*: hybrid intra-inter.

All the models are of 256 dimensions except Google W2V (300 dimensions). Numbers inside parenthesis indicate top-2 evaluation accuracy; Numbers outside parenthesis represent top-1 evaluation accuracy. Google Word2Vec, Word2Vec Groundtruth (trained on in-domain) and Baseline Word2Vec (trained on ASR transcriptions) perform better with the Semantic&Syntactic tasks, but fares poorly with acoustic analogy task. Intra-confusion performs well on acoustic analogy task while compromising on Semantic&Syntactic task. Inter-confusion performs well on both the acoustic analogy and Semantic&Syntactic tasks. Hybrid intra-inter training performs fairly well on all the three analogy tasks (acoustic, Semantic&Syntactic and Semantic&Syntactic–acoustic).

**Table A2:** Similarity task results: different proposed models.

Model	Similarity tasks	
	Word similarity	Acoustic similarity
Google W2V	0.6893 (7.9*e*-48)	−0.3489 (2.2*e*-28)
In-domain W2V	0.5794 (4.2*e*-29)	−0.2444 (1*e*-10)
C2V-1	0.4992 (3.3*e*-22)	0.1944 (1.7*e*-9)
C2V-a	0.105 (0.056)	0.8138 (5.1*e*-224)
C2V-c	0.2937 (5.4*e*-8)	0.8055 (5.1*e*-216)
C2V-*	0.0963 (0.08)	0.7858 (1.5*e*-198)

**Notes:**

C2V-1, top-confusion; C2V-a, intra-confusion; C2V-c, inter-confusion; C2V-*, hybrid intra-inter.

Similarity in terms of Spearman’s correlation. All the models are of 256 dimensions except Google W2V (300 dimensions). Numbers inside parenthesis indicate correlation *p*-value for similarity tasks Google Word2Vec, Baseline Word2Vec, and Word2Vec Groundtruth, all show high correlations with word similarity, while showing poor correlations on acoustic similarity. Google Word2Vec and Word2Vec Groundtruth models trained on clean data exhibit negative acoustic similarity correlation. Baseline Word2Vec trained on noisy ASR shows a small positive acoustic similarity correlation. Intra-confusion, inter-confusion, and hybrid intra-inter training show higher correlations on acoustic similarity.

**Table A3:** Analogy task results with Semantic&Syntactic splits: model pre-training/initialization.

Model	Analogy tasks							
	Semantic&Syntactic analogy			Acoustic analogy	Semantic&Syntactic–acoustic analogy			Average accuracy
	Semantic	Syntactic	Semantic&Syntactic		Semantic–acoustic	Syntactic–acoustic	Semantic&Syntactic–acoustic	
Google W2V	28.98% (35.75%)	70.79% (78.74%)	61.42% (69.1%)	0.9% (1.42%)	6.54% (14.38%)	17.9% (27.46%)	16.99% (26.42%)	26.44% (32.31%)
In-domain W2V	32.72% (39.99%)	66.53% (75.97%)	59.17% (68.14%)	0.6% (0.96%)	10.52% (17.46%)	10.5% (17.69%)	8.15% (13.5%)	22.64% (27.53%)
C2V-1	34.92% (41.96%)	68.7% (78.82%)	61.13% (70.56%)	0.9% (1.46%)	14.38% (19.28%)	16.85% (24.25%)	16.66% (23.86%)	26.23% (31.96%)
C2V-a	11.5% (15.53%)	67.56% (77.96%)	54.99% (63.97%)	9.04% (16.92%)	7.84% (10.46%)	36.92% (46.17%)	34.61% (43.34%)	32.88% (41.41%)
C2V-c	25.77% (33.12%)	60.1% (74.79%)	52.4% (65.45%)	16.54% (27.33%)	10.78% (14.05%)	28.9% (40.38%)	27.46% (38.29%)	32.13% (43.69%)
C2V-*	15.64% (21.94%)	66.73% (77.68%)	55.28% (65.19%)	10.49% (20.35%)	6.86% (11.11%)	35.4% (44.85%)	33.13% (42.18%)	36.27% (42.57%)

**Notes:**

C2V-1, top-confusion; C2V-a, intra-confusion; C2V-c, inter-confusion; C2V-*, hybrid intra-inter.

All the models are of 300 dimensions. Numbers inside parenthesis indicate top-2 evaluation accuracy; Numbers outside parenthesis represent top-1 evaluation accuracy. Pre-training is helpful in all the cases. Pre-training boosts the Semantic&Syntactic analogy accuracy for all. For intra-confusion, inter-confusion and hybrid intra-inter models, pre-training boosts the Semantic&Syntactic–acoustic analogy accuracies. A small dip in acoustic analogy accuracies is observed. However, overall average accuracy is improved.

**Table A4:** Similarity task results: model pre-training/initialization.

Model	Similarity tasks	
	Word similarity	Acoustic similarity
Google W2V	0.6893 (7.9*e*-48)	−0.3489 (2.2*e*-28)
In-domain W2V	0.4417 (3.5*e*-16)	−0.4377 (3.6*e*-33)
C2V-1	0.6036 (3.8*e*-34)	−0.4327 (2.5*e*-44)
C2V-a	0.5228 (1.4*e*-24)	0.62 (2.95*e*-101)
C2V-c	0.5798 (4.9*e*-31)	0.5825 (9.1*e*-87)
C2V-*	0.5341 (9.8*e*-26)	0.6237 (8.8*e*-103)

**Notes:**

C2V-1, top-confusion; C2V-a, intra-confusion; C2V-c, inter-confusion; C2V-*, hybrid intra-inter.

Similarity in terms of Spearman’s correlation. All the models are of 300 dimensions. Numbers inside parenthesis indicate correlation *p*-value for similarity tasks. Pre-training boosts the word similarity correlation for all the models. The correlation is improved considerably in the case of intra-confusion, inter-confusion, and hybrid intra-inter models while maintaining good correlation on acoustic similarity.

**Table A5:** Analogy task results: model concatenation and joint optimization.

Model	Fine-tuning	Analogy tasks							
		Semantic&Syntactic analogy			Acoustic analogy	Semantic&Syntactic–acoustic analogy			Average accuracy
	Scheme	Semantic	Syntactic	Semantic&Syntactic		Semantic–acoustic	Syntactic–acoustic	Semantic&Syntactic–acoustic	
Google W2V	–	28.98% (35.75%)	70.79% (78.74%)	61.42% (69.1%)	0.9% (1.42%)	6.54% (14.38%)	17.9% (27.46%)	16.99% (26.42%)	26.44% (32.31%)
In-domain W2V (556 dim.)	–	39.11% (48.03%)	70.41% (79.54%)	63.6% (72.69%)	0.81% (1.0%)	12.07% (18.62%)	14.79% (24.91%)	14.54% (24.33%)	26.32% (32.67%)
**Model concatenation**									
C2V-1 (F) + C2V-a (F)	–	6.22% (9.5%)	71.03% (83.65%)	56.51% (67.03%)	13.59% (25.43%)	6.54% (11.76%)	33.91% (42.82%)	31.74% (40.36%)	33.95% (44.27%)
C2V-1 (F) + C2V-c (F)	–	36.53% (47.01%)	57.94% (77.72%)	53.14% (70.84%)	20.99% (35.25%)	10.46% (16.01%)	26.31% (36.83%)	25.05% (35.18%)	33.06% (47.09%)
C2V-1 (F) + C2V-* (F)	–	11.85% (17.32%)	71.85% (82.74%)	58.4% (68.08%)	6.35% (11.39%)	7.84% (12.18%)	34.38% (43.78%)	32.28% (41.3%)	32.34% (40.26%)
**Fixed contextual subspace joint optimization**									
C2V-1 (F) + C2V-a (L)	Inter	22.96% (32.42%)	66.19% (82.98%)	56.5% (71.65%)	12.73% (20.54%)	13.4% (18.3%)	26.22% (35.09%)	25.21% (33.76%)	31.48% (41.98%)
C2V-1 (F) + C2V-a (L)	Intra	6.69% (11.58%)	69.79% (83.48%)	55.65% (67.37%)	17.03% (28.64%)	8.17% (13.73%)	31.85% (47.64%)	29.97% (39.09%)	34.22% (45.03%)
C2V-1 (F) + C2V-a (L)	Hybrid	11.69% (19.79%)	69.31% (84.53%)	56.39% (70.02%)	14.86% (25.84%)	9.8% (16.67%)	30.02% (38.94%)	28.42% (37.18%)	33.22% (44.35%)
C2V-1 (F) + C2V-c (L)	Inter	39.19% (50.57%)	58.35% (78.21%)	54.05% (72.01%)	23.33% (35.25%)	12.42% (18.3%)	24.45% (34.89%)	23.5% (33.58%)	33.63% (46.95%)
C2V-1 (F) + C2V-c (L)	Intra	22.76% (32.85%)	62.07% (80.34%)	53.26% (69.7%)	24.76% (39.32%)	7.52% (11.11%)	29.97% (41.47%)	28.19% (39.07%)	35.40% (49.36%)
C2V-1 (F) + C2V-c (L)	Hybrid	30.54% (43.21%)	61.56% (80.81%)	54.61% (72.38%)	23.6% (37.75%)	8.5% (14.71%)	28.25% (39.95%)	26.68% (37.95%)	34.96% (49.36%)
C2V-1 (F) + C2V-* (L)	Inter	27.02% (35.9%)	67.52% (81.6%)	58.45% (71.36%)	5.04% (8.55%)	11.76% (16.67%)	26.28% (34.64%)	25.13% (33.21%)	29.54% (37.71%)
C2V-1 (F) + C2V-* (L)	Intra	10.48% (15.84%)	70.44% (81.57%)	57.00% (66.85%)	7.21% (13.33%)	6.21% (12.09%)	34.07% (42.52%)	31.87% (40.1%)	32.03% (40.09)
C2V-1 (F) + C2V-* (L)	Hybrid	15.41% (23.31%)	70.56% (82.61%)	58.2% (68.32%)	6.39% (11.61%)	8.17% (12.09%)	32.36% (40.43%)	30.44% (38.19%)	31.68% (39.37%)
**Unrestricted joint optimization**									
C2V-1 (L) + C2V-a (L)	Inter	8.6% (14.74%)	57.96% (75.8%)	46.9% (62.12%)	30.73% (46.42%)	5.88% (12.75%)	26.79% (38.44%)	25.13% (36.4%)	34.25% (48.31%)
C2V-1 (L) + C2V-a (L)	Intra	4.97% (7.9%)	69.27% (81.30%)	54.86% (64.85%)	23.86% (40.55%)	7.84% (11.44%)	34.92% (45.02%)	32.77% (42.38%)	37.16% (49.26%)
C2V-1 (L) + C2V-a (L)	Hybrid	1.1% (1.64%)	26.54% (40.32%)	20.83% (31.65%)	49.25% (61.91%)	2.29% (3.92%)	15.05% (25.24%)	14.04% (23.55%)	28.12% (39.04%)
C2V-1 (L) + C2V-c (L)	Inter	33.01% (43.72%)	50.81% (71.13%)	46.82% (64.98%)	37.15% (52.99%)	9.48% (16.01%)	23.16% (36.41%)	22.07% (34.79%)	35.35% (50.92%)
C2V-1 (L) + C2V-c (L)	Intra	21.9% (30.43%)	58.99% (76.12%)	50.68% (65.88%)	33.05% (49.4%)	7.52% (10.46%)	31.23% (44.12%)	29.35% (41.51%)	37.69% (52.26%)
C2V-1 (L) + C2V-c (L)	Hybrid	10.48% (15.72%)	30.0% (44.25%)	25.63% (37.86%)	52.73% (67.21%)	3.27% (4.9%)	16.09% (27.77%)	15.08% (25.96%)	31.15% (43.68%)
C2V-1 (L) + C2V-* (L)	Inter	19.24% (26.59%)	61.57% (76.8%)	52.08% (65.54%)	17.85% (27.97%)	7.52% (12.75%)	28.81% (38.94%)	27.12% (36.87%)	32.35% (43.46%)
C2V-1 (L) + C2V-* (L)	Intra	10.09% (13.77%)	68.76% (79.06%)	55.61% (64.42%)	10.34% (20.05%)	5.88% (9.48%)	36.13% (45.41%)	33.73% (42.56%)	33.23% (42.34%)
C2V-1 (L) + C2V-* (L)	Hybrid	12.98% (17.91%)	68.26% (79.62%)	55.87% (65.79%)	11.73% (22.63%)	5.88% (10.46%)	35.28% (43.92%)	32.95% (41.3%)	33.52% (43.24%)

**Notes:**

C2V-1, top-confusion; C2V-a, intra-confusion; C2V-c, inter-confusion; C2V-*, hybrid intra-inter.

Numbers inside parenthesis indicate top-2 evaluation accuracy; All the models are of 556 dimensions. Numbers outside parenthesis represent top-1 evaluation accuracy. Acronyms: (F):Fixed embedding, (L):Learn embedding during joint training. Model concatenation provides gains in acoustic-analogy task and thereby resulting in gains in average accuracy compared to results in Table A3 for intra-confusion and inter-confusion models. Fixed contextual subspace and unrestricted joint optimizations further improves results over model concatenation. Best results in terms of average accuracy is obtained with unrestricted joint optimizations, an absolute improvement of 10%. Confusion2Vec models surpass Word2Vec even for Semantic&Syntactic analogy task (top-2 evaluation accuracy).

**Table A6:** Similarity task results: model concatenation and joint optimization.

Model	Fine-tuning scheme	Similarity tasks	
		Word similarity	Acoustic similarity
Google W2V	–	0.6893 (7.9*e*-48)	−0.3489 (2.2*e*-28)
In-domain W2V (556 dim.)	–	0.6333 (4.9*e*-36)	−0.4717 (5.7*e*-39)
**Model concatenation**			
C2V-1 (F) + C2V-a (F)	–	0.5102 (2.9*e*-23)	0.7231 (2.2*e*-153)
C2V-1 (F) + C2V-c (F)	–	0.5609 (9.8*e*-29)	0.6345 (2.3*e*-107)
C2V-1 (F) + C2V-* (F)	–	0.4142 (4.1*e*-15)	0.5285 (5.6*e*-69)
**Fixed contextual subspace joint optimization**			
C2V-1 (F) + C2V-a (L)	Inter	0.5676 (1.6*e*-29)	0.4437 (9.1*e*-47)
C2V-1 (F) + C2V-a (L)	Intra	0.5211 (2.3*e*-24)	0.6967 (6.5*e*-138)
C2V-1 (F) + C2V-a (L)	Hybrid	0.5384 (3.4*e*-26)	0.6287 (6.7*e*-105)
C2V-1 (F) + C2V-c (L)	Inter	0.5266 (6.1*e*-25)	0.5818 (1.6*e*-86)
C2V-1 (F) + C2V-c (L)	Intra	0.5156 (8.3*e*-24)	0.7021 (6.3*e*-141)
C2V-1 (F) + C2V-c (L)	Hybrid	0.5220 (1.8*e*-24)	0.6674 (1.4*e*-122)
C2V-1 (F) + C2V-* (L)	Inter	0.5587 (1.7*e*-28)	0.302 (2.5*e*-21)
C2V-1 (F) + C2V-* (L)	Intra	0.4996 (3.1*e*-22)	0.5691 (4.7*e*-82)
C2V-1 (F) + C2V-* (L)	Hybrid	0.5254 (8.2*e*-25)	0.4945 (2.6*e*-59)
**Unrestricted joint optimization**			
C2V-1 (L) + C2V-a (L)	Inter	0.5513 (1.3*e*-27)	0.7926 (2.4*e*-204)
C2V-1 (L) + C2V-a (L)	Intra	0.5033 (1.4*e*-22)	0.7949 (2*e*-206)
C2V-1 (L) + C2V-a (L)	Hybrid	0.1067 (0.0528)	0.8309 (8.5*e*-242)
C2V-1 (L) + C2V-c (L)	Inter	0.5763 (1.3*e*-30)	0.7725 (8.2*e*-188)
C2V-1 (L) + C2V-c (L)	Intra	0.5379 (3.8*e*-26)	0.7717 (3.5*e*-187)
C2V-1 (L) + C2V-c (L)	Hybrid	0.2295 (2.6*e*-5)	0.8294 (3.6*e*-240)
C2V-1 (L) + C2V-* (L)	Inter	0.5338 (1*e*-25)	0.6953 (3.7*e*-137)
C2V-1 (L) + C2V-* (L)	Intra	0.4920 (1.6*e*-21)	0.6942 (1.5*e*-136)
C2V-1 (L) + C2V-* (L)	Hybrid	0.4967 (5.8*e*-22)	0.6986 (5.9*e*-139)

**Notes:**

C2V-1, top-confusion; C2V-a, intra-confusion; C2V-c, inter-confusion; C2V-*, hybrid intra-inter.

Similarity in terms of Spearman’s correlation. All the models are of 556 dimensions. Numbers inside parenthesis indicate correlation *p*-value for similarity tasks. Good correlations are observed for both the word similarity and acoustic similarity with model concatenation with and without joint optimization. All the correlations are found to be statistically significant.

## Additional Information and Declarations

### Data Availability

The following information was supplied regarding data availability: Data are derivatives of LDC datasets: https://www.ldc.upenn.edu and derived using standard Kaldi recipes as described in the article: http://kaldi-asr.org. Our training and validation code is at: https://bitbucket.org/georgiou/confusion2vec.

## References

[ref-1] Abadi M, Barham P, Chen J, Chen Z, Davis A, Dean J, Devin M, Ghemawat S, Irving G, Isard M, Kudlur M, Levenberg J, Monga R, Moore S, Murray DG, Steiner B, Tucker P, Vasudevan V, Warden P, Wicke M, Yu Y, Zheng X (2016). Tensorflow: a system for large-scale machine learning.

[ref-2] Allauzen A (2007). Error detection in confusion network.

[ref-3] Bengio Y, Ducharme R, Vincent P, Jauvin C (2003). A neural probabilistic language model. Journal of Machine Learning Research.

[ref-4] Bengio S, Heigold G (2014). Word embeddings for speech recognition.

[ref-5] Bertoldi N, Zens R, Federico M (2007). Speech translation by confusion network decoding.

[ref-6] Blei DM, Ng AY, Jordan MI (2003). Latent dirichlet allocation. Journal of Machine Learning Research.

[ref-7] Bojanowski P, Grave E, Joulin A, Mikolov T (2017). Enriching word vectors with subword information. Transactions of the Association for Computational Linguistics.

[ref-8] Botha JA, Blunsom P (2014). Compositional morphology for word representations and language modelling.

[ref-9] Buckman J, Neubig G (2018). Neural lattice language models. Transactions of the Association for Computational Linguistics.

[ref-10] Celebi A, Sak H, Dikici E, Saraçlar M, Lehr M, Prud’hommeaux E, Xu P, Glenn N, Karakos D, Khudanpur S, Roark B, Sagae K, Shafran I, Bikel D, Callison-Burch C, Cao Y, Hall K, Hasler E, Koehn P, Lopez A, Post M, Riley D (2012). Semi-supervised discriminative language modeling for Turkish ASR.

[ref-11] Chelba C, Hazen TJ, Saraclar M (2008). Retrieval and browsing of spoken content. IEEE Signal Processing Magazine.

[ref-12] Chen X, Xu L, Liu Z, Sun M, Luan H (2015). Joint learning of character and word embeddings.

[ref-13] Chung Y-A, Wu C-C, Shen C-H, Lee H-Y, Lee L-S (2016). Audio word2vec: unsupervised learning of audio segment representations using sequence-to-sequence autoencoder.

[ref-14] Cieri C, Miller D, Walker K (2004). The fisher corpus: a resource for the next generations of speech-to-text.

[ref-15] Costa-Jussà MR, Fonollosa JAR (2007). Analysis of statistical and morphological classes to generate weighted reordering hypotheses on a statistical machine translation system.

[ref-16] Cotterell R, Schütze H (2015). Morphological word-embeddings.

[ref-17] Deerwester S, Dumais ST, Furnas GW, Landauer TK, Harshman R (1990). Indexing by latent semantic analysis. Journal of the American Society for Information Science.

[ref-18] Dikici E, Celebi A, Saraçlar M (2012). Performance comparison of training algorithms for semi-supervised discriminative language modeling.

[ref-19] Dyer CJ (2007). The “noisier channel”: translation from morphologically complex languages.

[ref-20] Dyer C (2009). Using a maximum entropy model to build segmentation lattices for MT.

[ref-21] Dyer CJ (2010). A formal model of ambiguity and its applications in machine translation.

[ref-22] Dyer C, Muresan S, Resnik P (2008). Generalizing word lattice translation.

[ref-23] Erhan D, Bengio Y, Courville A, Manzagol P-A, Vincent P, Bengio S (2010). Why does unsupervised pre-training help deep learning?. Journal of Machine Learning Research.

[ref-24] Faruqui M, Dyer C (2014). Improving vector space word representations using multilingual correlation.

[ref-25] Finkelstein L, Gabrilovich E, Matias Y, Rivlin E, Solan Z, Wolfman G, Ruppin E (2001). Placing search in context: the concept revisited. Proceedings of the 10th International Conference on World Wide Web.

[ref-26] Ghannay S, Estève Y, Camelin N (2015a). Word embeddings combination and neural networks for robustness in ASR error detection.

[ref-27] Ghannay S, Estève Y, Camelin N, Deléglise P (2016). Acoustic word embeddings for ASR error detection.

[ref-28] Ghannay S, Estève Y, Camelin N, Dutrey C, Santiago F, Adda-Decker M (2015b). Combining continuous word representation and prosodic features for ASR error prediction.

[ref-29] Hakkani-Tür D, Béchet F, Riccardi G, Tur G (2006). Beyond ASR 1-best: using word confusion networks in spoken language understanding. Computer Speech & Language.

[ref-30] Hardmeier C, Bisazza A, Federico M (2010). FBK at WMT 2010: word lattices for morphological reduction and chunk-based reordering.

[ref-31] He W, Wang W, Livescu K (2016). Multi-view recurrent neural acoustic word embeddings.

[ref-32] Hofmann T (1999). Probabilistic latent semantic analysis.

[ref-33] Hoffmeister B, Hillard D, Hahn S, Schluter R, Ostendor M, Ney H (2007). Cross-site and intra-site ASR system combination: comparisons on lattice and 1-best methods.

[ref-34] Hori T, Hetherington IL, Hazen TJ, Glass JR (2007). Open-vocabulary spoken utterance retrieval using confusion networks.

[ref-35] Huang EH, Socher R, Manning CD, Ng AY (2012). Improving word representations via global context and multiple word prototypes.

[ref-36] Jiang H (2005). Confidence measures for speech recognition: a survey. Speech Communication.

[ref-37] Joulin A, Grave E, Bojanowski P, Mikolov T (2016). Bag of tricks for efficient text classification.

[ref-38] Kamper H, Wang W, Livescu K (2016). Deep convolutional acoustic word embeddings using word-pair side information.

[ref-39] Kemp T, Schaaf T (1997). Estimating confidence using word lattices.

[ref-40] Kim Y (2014). Convolutional neural networks for sentence classification. arXiv preprint.

[ref-41] Kruengkrai C, Uchimoto K, Kazama J, Wang Y, Torisawa K, Isahara H (2009). An error-driven word-character hybrid model for joint Chinese word segmentation and POS tagging.

[ref-42] Kurata G, Itoh N, Nishimura M (2011). Training of error-corrective model for ASR without using audio data.

[ref-43] Ladhak F, Gandhe A, Dreyer M, Mathias L, Rastrow A, Hoffmeister B (2016). Latticernn: recurrent neural networks over lattices.

[ref-44] Lavie A, Waibel A, Levin L, Finke M, Gates D, Gavalda M, Zeppenfeld T, Zhan P (1997). Janus-iii: Speech-to-speech translation in multiple languages.

[ref-45] Le Q, Mikolov T (2014). Distributed representations of sentences and documents.

[ref-46] Levin K, Henry K, Jansen A, Livescu K (2013). Fixed-dimensional acoustic embeddings of variable-length segments in low-resource settings.

[ref-47] Levy O, Goldberg Y (2014). Dependency-based word embeddings.

[ref-48] Lilleberg J, Zhu Y, Zhang Y (2015). Support vector machines and word2vec for text classification with semantic features.

[ref-49] Ling W, Dyer C, Black AW, Trancoso I (2015). Two/too simple adaptations of word2vec for syntax problems.

[ref-50] Liu X, Wang Y, Chen X, Gales MJF, Woodland PC (2014). Efficient lattice rescoring using recurrent neural network language models.

[ref-51] Luong T, Socher R, Manning C (2013). Better word representations with recursive neural networks for morphology.

[ref-52] Mangu L, Brill E, Stolcke A (2000). Finding consensus in speech recognition: word error minimization and other applications of confusion networks. Computer Speech & Language.

[ref-53] Marin A, Kwiatkowski T, Ostendorf M, Zettlemoyer L (2012). Using syntactic and confusion network structure for out-of-vocabulary word detection.

[ref-54] Mathias L, Byrne W (2006). Statistical phrase-based speech translation.

[ref-55] Matusov E, Kanthak S, Ney H (2005). On the integration of speech recognition and statistical machine translation.

[ref-56] Matusov E, Ueffing N, Ney H (2006). Computing consensus translation for multiple machine translation systems using enhanced hypothesis alignment.

[ref-57] Mikolov T, Chen K, Corrado G, Dean J (2013a). Efficient estimation of word representations in vector space.

[ref-58] Mikolov T, Karafiát M, Burget L, Černockỳ J, Khudanpur S (2010). Recurrent neural network based language model.

[ref-59] Mikolov T, Le QV, Sutskever I (2013b). Exploiting similarities among languages for machine translation. arXiv preprint.

[ref-60] Mikolov T, Sutskever I, Chen K, Corrado GS, Dean J (2013c). Distributed representations of words and phrases and their compositionality.

[ref-61] Mnih A, Kavukcuoglu K (2013). Learning word embeddings efficiently with noise-contrastive estimation.

[ref-62] Niehues J, Kolss M (2009). A POS-based model for long-range reorderings in SMT.

[ref-63] Ogata J, Goto M (2005). Speech repair: quick error correction just by using selection operation for speech input interfaces.

[ref-64] Onishi T, Utiyama M, Sumita E (2011). Paraphrase lattice for statistical machine translation. IEICE Transactions on Information and Systems.

[ref-65] Pennington J, Socher R, Manning C (2014). Glove: global vectors for word representation.

[ref-66] Povey D, Ghoshal A, Boulianne G, Burget L, Glembek O, Goel N, Hannemann M, Motlicek P, Qian Y, Schwarz P, Silovsky J, Stemmer G, Vesely K (2011). The kaldi speech recognition toolkit.

[ref-67] Qiu S, Cui Q, Bian J, Gao B, Liu T-Y (2014). Co-learning of word representations and morpheme representations.

[ref-68] Quirk C, Brockett C, Dolan W (2004). Monolingual machine translation for paraphrase generation.

[ref-69] Rosti A-V, Ayan NF, Xiang B, Matsoukas S, Schwartz R, Dorr B (2007a). Combining outputs from multiple machine translation systems.

[ref-70] Rosti A-V, Matsoukas S, Schwartz R (2007b). Improved word-level system combination for machine translation.

[ref-71] Sagae K, Lehr M, Prud’hommeaux E, Xu P, Glenn N, Karakos D, Khudanpur S, Roark B, Saraclar M, Shafran I, Bikel D, Callison-Burch C, Cao Y, Hall K, Hasler E, Koehn P, Lopez A, Post M, Riley D (2012). Hallucinated n-best lists for discriminative language modeling.

[ref-72] Schnabel T, Labutov I, Mimno D, Joachims T (2015). Evaluation methods for unsupervised word embeddings.

[ref-73] Schroeder J, Cohn T, Koehn P (2009). Word lattices for multi-source translation.

[ref-74] Schultz T, Jou S-C, Vogel S, Saleem S (2004). Using word latice information for a tighter coupling in speech translation systems.

[ref-75] Seigel MS, Woodland PC (2011). Combining information sources for confidence estimation with CRF models.

[ref-76] Shivakumar PG, Li H, Knight K, Georgiou P (2018). Learning from past mistakes: improving automatic speech recognition output via noisy-clean phrase context modeling. APSIPA Transactions on Signal and Information Processing.

[ref-77] Soricut R, Och F (2015). Unsupervised morphology induction using word embeddings.

[ref-78] Sperber M, Neubig G, Niehues J, Waibel A (2017). Neural lattice-to-sequence models for uncertain inputs.

[ref-79] Su J, Tan Z, Xiong D, Ji R, Shi X, Liu Y (2017). Lattice-based recurrent neural network encoders for neural machine translation.

[ref-80] Sundermeyer M, Tüske Z, Schlüter R, Ney H (2014). Lattice decoding and rescoring with long-span neural network language models.

[ref-81] Tai KS, Socher R, Manning CD (2015). Improved semantic representations from tree-structured long short-term memory networks.

[ref-82] Tan QF, Audhkhasi K, Georgiou PG, Ettelaie E, Narayanan SS (2010). Automatic speech recognition system channel modeling.

[ref-83] Tan Z, Su J, Wang B, Chen Y, Shi X (2018). Lattice-to-sequence attentional neural machine translation models. Neurocomputing.

[ref-84] Tur G, Wright J, Gorin A, Riccardi G, Hakkani-Tür D (2002). Improving spoken language understanding using word confusion networks.

[ref-85] Wahlster W (2013). Verbmobil: foundations of speech-to-speech translation.

[ref-86] Weide R (1998). The CMU pronunciation dictionary, release 0.6. http://www.speech.cs.cmu.edu/cgi-bin/cmudict.

[ref-87] Wuebker J, Ney H (2012). Phrase model training for statistical machine translation with word lattices of preprocessing alternatives.

[ref-88] Xing C, Wang D, Zhang X, Liu C (2014). Document classification with distributions of word vectors.

[ref-89] Xiong W, Droppo J, Huang X, Seide F, Seltzer M, Stolcke A, Yu D, Zweig G (2016). Achieving human parity in conversational speech recognition. arXiv preprint.

[ref-90] Xu H, Povey D, Mangu L, Zhu J (2011). Minimum bayes risk decoding and system combination based on a recursion for edit distance. Computer Speech & Language.

[ref-91] Xu P, Roark B, Khudanpur S (2012). Phrasal cohort based unsupervised discriminative language modeling.

[ref-92] Xue J, Zhao Y (2005). Improved confusion network algorithm and shortest path search from word lattice.

[ref-93] Yin W, Schütze H (2016). Learning word meta-embeddings. http://aclweb.org/anthology/P/P16/P16-1128.pdf.

